# Rapid Deposition of Layered Polymer Films by Ring-Opening Metathesis Polymerization during Spin Coating

**DOI:** 10.1021/acsapm.6c01078

**Published:** 2026-06-23

**Authors:** Matthew P. Vasuta, Skyler T. Hornback, G. Kane Jennings

**Affiliations:** † Interdisciplinary Materials Science Program, 5718 Vanderbilt University, Nashville, Tennessee 37235, United States; ‡ Department of Chemical and Biomolecular Engineering, Vanderbilt University, Nashville, Tennessee 37235, United States

**Keywords:** ROMP, layered films, spin coating, wetting, protein resistance

## Abstract

The surface composition of a polymer thin film determines the interactions of the film with its environment and equips the film for specific applications, such as a coating, sensor, adhesive, antifoulant, or membrane. We report a rapid and versatile method to tailor the surface properties of polymer films by growing functional top layers on top of inexpensive bottom layers through sequential depositions of different monomer solutions onto a catalyst-coated surface. This method of synthesizing layered films formally expands spin coating ring-opening metathesis polymerization (scROMP), which efficiently integrates polymer film synthesis and deposition into one rapid process, converting the sequential monomers into layered polymer films in under 3 min with less than 600 μL of solvent for a 2.25 cm^2^ film. The scROMP approach is shown here to synthesize films with polymers of >500 kDa molecular weights and <1.15 dispersities and to effectively stack two or more layers of distinct functionalities that constitute the bulk and surface regions of the film. Thicknesses of the top layers can be dramatically altered by monomer concentration, and the top layer can be extremely thin (within the nanometer range) or as thick as 3× the bottom layer (>15 μm) for certain compositions. As potential applications, we demonstrate the syntheses of unique layered films with specialized properties such as omniphobicity, superhydropilicity, and protein resistance.

## Graphical abstract

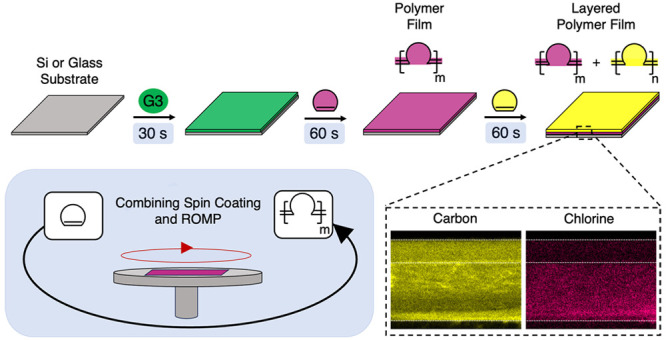

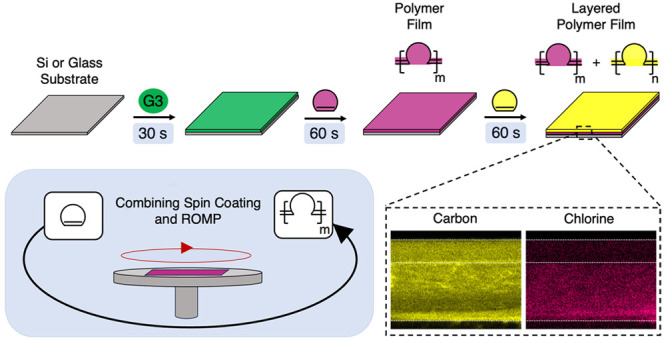

## Introduction

The surface of a polymer film impacts the performance of the film in many applications despite composing only a slight fraction of the overall film.^1,2^ When an application requires a highly specialized surface that must be synthesized with expensive monomer, localizing the pricey polymer on the surface atop an inexpensive lower structural layer is often advantageous. Herein, we report a combined polymerization and deposition method that synthesizes layers of targeted functionality atop less expensive bottom layers both faster and with fewer steps than traditional methods for layered film synthesis. This method, an expansion of spin coating ring-opening metathesis polymerization (scROMP),^3^ operates by leveraging the kinetically rapid and living nature of ROMP catalyzed by Grubbs third generation catalyst^4,5^ to simultaneously deposit and polymerize monomer into layered polymer films. We demonstrate here that scROMP can synthesize 8–30 μm thick layered films with chains of molecular weight >500 kDa and dispersities <1.15 in <3 min using <600 μL of solvent; additionally, by postpolymerization modification, top layers of varied surface energies and protein resistances are also easily attainable to design layered films for specific applications.

The rapid synthesis time and overall synthetic simplicity of scROMP presents a distinctive shift from current fabrication methods for layered polymer films described within the literature. Layer-by-layer (LbL) assembly is an attractive method to deposit two or more layers of distinct functionality because of the method’s lack of substrate geometrical requirements and similar design mimicry to biological media.^6^ The process, however, is limited to charged polyions and is not rapid, requiring many hours and deposition steps to synthesize films on the scale of a few hundred nanometers.^7,8^ Sun et al. showed that manipulation of process conditions to obtain LbL films with thicknesses in the micron-range caused layers to delaminate from each other, which significantly decreased the membrane performance of the micron-thick films relative to thinner LbL counterparts.^9^ One approach to circumvent these issues related to thickness and requirement of charge is to instead synthesize the polymers in solution and then successively spin coat the distinct polymer layers on top of each other. Several studies show thicknesses for spin coated films in the micron-range, and thicknesses can be controlled relatively easily by varying spin speeds during deposition.^10^ However, this process still remains time- and resource-intensive as it requires solution polymerization and associated separations, which greatly elevate bulk organic solvent usage, and from a performance standpoint, poor adhesion between chemically dissimilar layers may cause delamination.^11,12^

Beyond the layering of two or more separate polymer species together, the copolymerization of distinct species as a block copolymer brush is a viable method to synthesize distinctly layered functionality. “Grafting-to” methods are often used to attach presynthesized block copolymers to a substrate, but because each chain must essentially diffuse through the growing film and tether to a reactive site on the substrate, film thicknesses rarely exceed 30 nm.^13^ “Grafting-from” or surface-initiated approaches are widely employed because of their ability to fabricate much thicker robustly bound block copolymer brushes with thicknesses of tens of nanometers to up to the micron scale.^14^ The most popular surface-initiated methods for block polymer brushes include atom transfer radical polymerization (ATRP),^15^ reversible addition–fragmentation chain transfer (RAFT),^16^ nitroxide-mediated polymerization (NMP),^17^ and photoiniferter-mediated polymerization (PIMP),^18^ and numerous examples can be found throughout the literature using these methods to synthesize poly­(methacrylate)-,^19,20^ poly­(acrylate)-,^21,22^ poly­(acrylamide)-,^23,24^ poly­(styrene)-,^25,26^ and poly­(vinylpyridine)-based^27,28^ top blocks on bottom blocks of different compositions. Grafting-from syntheses often require stringent reaction conditions and long reaction times, however. The most utilized surface-initiated synthesis method for block copolymer films, ATRP, typically requires hours to functionalize the substrate with the chosen initiator, several freeze–pump–thaw degassing cycles for the monomer solution, and then multiple hours of synthesis time for each block.^29,30^ Surface-initiated polymerizations are also often limited in their outer block thicknesses due to decreasing reinitiation efficiencies.^31^ Additionally, the usually advantageous feature of covalent bonding to the substrate may pose challenges from a materials discovery perspective where many characterization techniques require removal of the copolymer from the substrate and dissolution into an appropriate solvent.

Here, we present a method to generate layered polymer films by growing a functional polymer atop an existing inexpensive polymer film through combined spin coating and polymerization. We achieve this coupling of polymers by expanding upon the spin coating ring-opening metathesis polymerization (scROMP) method that we previously reported.^3^ The scROMP method has been demonstrated to synthesize homopolymer and random copolymer films via sequential additions of Grubbs third-generation catalyst and monomer onto a spinning substrate.^3^ Subsequent studies have demonstrated the synthesis of semifluorinated polymer thin films and membranes using scROMP^32^ and the effect of reducing per- and polyfluoroalkyl substances (PFAS) on the surface and membrane properties of those films via copolymerization with a hydrocarbon monomer.^33^ We expand here from previous studies by investigating the effects of sequential monomer depositions during spin coating. Since the polymer chains in scROMP grow through a living polymerization upward from the substrate,^3^ we show here that parallel-layered polymer thin films can be synthesized by an additional dispense of a different cyclic olefin monomer 60 s after the dispense of the first cyclic olefin monomer as shown in Scheme 1.

**Scheme 1:**
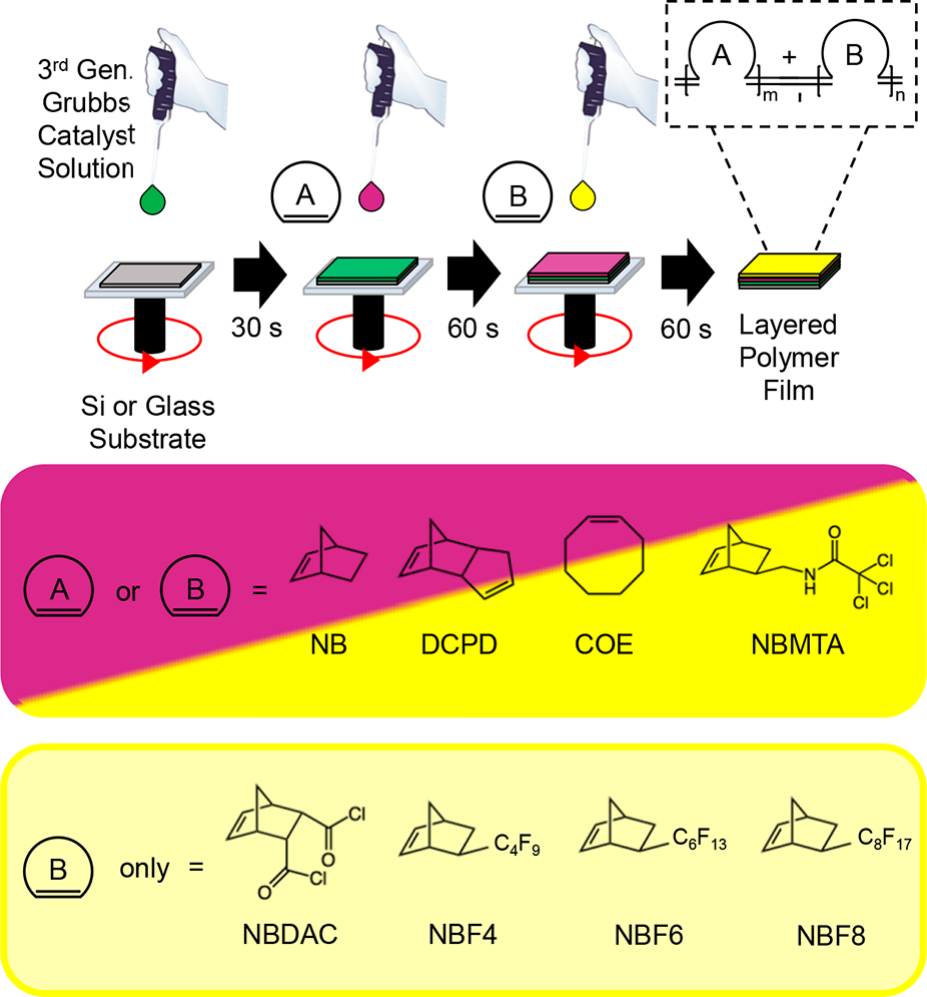
Synthetic Procedure of Spin Coating Ring-Opening Metathesis Polymerization for the Fabrication of Layered Polymer Thin Films from Two or More Cyclic Olefin Monomers

By using the scROMP method for the synthesis of layered films, we can vary the composition of the outer layer to achieve surfaces with coveted properties, such as low or high wettability to water or protein resistance, all built atop inexpensive but structurally supportive lower layers. We chose three inexpensive ROMP-active monomers to fabricate the bottom layers in this study: norbornene (NB), dicyclopentadiene (DCPD), and *cis*-cyclooctene (COE) (the fourth, 5-(*N*-methyltrichloroacetamide)­norbornene (NBMTA), was only used to generate a chlorine map signal in energy-dispersive X-ray spectroscopy). Norbornene is the nonfunctionalized form of a large class of ROMP-active norbornenyl-based monomers whose polymerized forms have been used extensively for gas separation,^34^ bioconjugation,^35^ or as optical waveguides.^36^ Dicyclopentadiene possesses a second olefin group along its cyclopentyl substituent that may undergo secondary reactions and form cross-links across polymer chains, making poly­(dicyclopentadiene) films tougher and more suitable for use as aerogels^37^ or body panels in farming equipment.^38^ *cis*-Cyclooctene polymerizes to form poly­(cyclooctene), which finds more targeted uses as a soft and tacky adhesive^39^ or as a toughening template for nanoporous polymer membranes.^40^ In this work, we demonstrate that other monomers can be polymerized on top of these structural base layers to generate functional top layers with surface compositions that are relevant to active areas of research. These syntheses are faster and contain fewer steps than many traditional methods for layered polymer film syntheses and should be suitable for future thin-film exploration.

## Experimental Section

### Materials and Methods

#### Materials

Grubbs catalyst (2nd generation) (1,3-bis­(2,4,6-trimethylphenyl)-2-(imidazolidinylidene)­(dichlorophenylmethylene)), hydroquinone, 3-bromopyridine, *trans*-3,6-endomethylene-1,2,3,6-tetrahydrophthaloyl chloride (norbornene di-acyl chloride [NBDAC]), *cis*-cyclooctene (COE), dicyclopentadiene (DCPD), bicyclo[2·2·1]­hept-2-ene (norbornene [NB]), ethylene glycol, diiodomethane, glycerol, *n*-hexane, l-lysine hydrate (97%), l-arginine, hydroxylamine hydrochloride, chloroform-d, trichloroacetyl chloride, pyridine, albumin-fluorescein isothiocyanate conjugate bovine (FITC-BSA), 3-(*N*-morpholino)­propanesulfonic acid (MOPS), and HPLC-grade (>99.9%) tetrahydrofuran (THF) were used as received from Sigma-Aldrich. 1H,1H,2H-Perfluoro-1-hexene (99%), 1H,1H,2H-perfluoro-1-octene (99%), dichloromethane (DCM) (99.8%), *n*-pentane (98%), *n*-decane (99+%), *n*-dodecane (99%), *n*-tetradecane (99+%), *n*-hexadecane (99%), and 75 × 38 × 1.0 mm plain microscope slides were obtained from Thermo Fisher Scientific. 1H,1H,2H-Perfluoro-1-decene (97%) and 5-norbornene-2-methylamine (NBNH_2_) were purchased from the Tokyo Chemical Industry Co., Ltd. Silicon (100) wafers were purchased from University Wafers. Deionized water (DI water) (16.7 MΩ·cm) was purified with a Modu-Pure system. Ethanol (200 proof) was obtained from Decon Laboratories. Polystyrene standards were obtained from Agilent.

#### Preparation of Silicon Substrates

Silicon (100) wafers were cut using a diamond scribe into 1.5 × 1.5 cm substrates, rinsed with DI water and ethanol, and dried in a nitrogen stream prior to use. All films except those used for confocal microscopy experiments were synthesized on silicon substrates.

#### Preparation of Glass Substrates

Plain microscope slides were cut using a diamond scribe into 1.5 cm × 1.5 cm substrates, rinsed with DI water and ethanol, and dried in a nitrogen stream prior to use in confocal microscopy.

#### Synthesis of 5-(Perfluoro-*n*-alkyl)­norbornene (NBF*n*) Monomers

5-(Perfluorobutyl)­norbornene (NBF4), 5-(perfluorohexyl)­norbornene (NBF6), and 5-(perfluorooctyl)­norbornene (NBF8) monomers were synthesized as previously reported.^41^ If the distillate product was cloudy, sequential aliquots of ethanol and then DI water of approximately half of the volume of the distillate product were added to the vial to partition the product mixture into a biphasic solution. The clear phase was collected and then further separated using a rotary evaporator to yield the NBF*n* product, which was previously reported by our group to give respective yields of 45%, 50%, and 43% based on ^1^H and ^19^F NMR analyses.^42^

#### Synthesis of 5-(*N*-Methyltrichloroacetamide)­norbornene (NBMTA) Monomer

1.1 mol equiv of trichloroacetyl chloride in DCM were added dropwise to a round-bottom flask containing 1 mol equiv of 5-norbornene-2-methylamine and 1.5 equiv of pyridine in DCM. The reaction took place overnight at 0 °C under a N_2_ atmosphere. The reaction product was washed sequentially with DI water, 1 M HCl_(aq)_, 2 M NaHCO_3(aq)_, and 4 M NaCl_(aq)_ and concentrated using a rotary evaporator. ^1^H NMR spectra in Figure S1 confirm successful modification of the norbornene methylamine to the NBMTA product.

#### Polymerization

Grubbs third generation catalyst (G3) was synthesized from Grubbs second generation catalyst and 3-bromopyridine using a procedure developed by Love et al.^4^ G3 is stable for long time periods in its solid form but loses its catalytic activity fairly rapidly when dissolved in solvent;^43^ as such, 5 mM of solid G3 was dissolved in DCM no more than 5 min before polymerization. To prepare the substrate for polymerization, 200 μL of the 5 mM G3 solution was spin coated at 2000 rpm. Next, 200 μL of a first monomer was dispensed on the spinning substrate and spin coated for 60 s at 2000 rpm. This was followed by dispensing 200 μL of a second type of monomer and an additional 60 s of spin coating at 2000 rpm. For three-layered systems, monomer dispenses were only 30 s apart from each other, but the last monomer was given 60 s to spin coat, similar to single-layer and two-layer systems.

NBF4, NBF6, NBF8, NBDAC, and COE monomers were all dispensed as neat liquids unless otherwise noted. Since NB, DCPD, and NBMTA are solids, NB was diluted to 2.7 M in *n*-pentane, DCPD was diluted to 4 M in DCM, and NBMTA was diluted to 4 M in DCM. For NMR characterizations, films were removed from the surface using a razor blade and dissolved for 1 h in 2 mL of chloroform-d. For GPC characterizations, films were similarly removed with a razor blade but required 1 h of heating at 60 °C in 2 mL of THF to dissolve. For protein adsorption experiments, films synthesized on glass substrates were placed in a solution containing 0.2 mg/mL of FITC-BSA in 50 mM MOPS_(aq)_ in the absence of light for 1 h, as was shown by de las Heras Alarcón et al.^44^ Films were rinsed with 50 mM MOPS_(aq)_ and water prior to confocal imaging.

#### Characterization

Scanning electron microscopy (SEM) images were obtained on a Zeiss Merlin SEM with an accelerating voltage of 15 kV. Energy dispersive X-ray spectroscopy (EDS) maps were obtained using an Everhart–Thornley detector with the beam current set to 1 nA. Samples were adhered at a 90° angle to a cross-sectional stub using double-sided carbon tape and were sputter coated with gold using a Cressington 108 Sputter Coater prior to imaging.

Gel permeation chromatography (GPC) analysis was performed using a Waters 1525 Binary High-Performance Liquid Chromatography (HPLC) pump system equipped with a Waters 2414 refractive index (RI) detector and in-line degasser. HPLC-grade THF was selected for the mobile phase and pumped at a flow rate of 1 mL/min through three Waters Styragel columns containing 5 μm diameter beads of 10,000, 1000, and 100 Å pore sizes at 30 °C. RI detection was performed at 30 °C at a sampling rate of 5 points/s, a filter time of 3.0 s, and a sensitivity of 7.81 × 10^–5^ RIU/V. Polystyrene standards (2.09 ≤ M_N_ ≤ 560 kDa) were used to generate a linear calibration curve for sample molecular weight and dispersity assignments.

Stylus profilometry was recorded on a Bruker Dektak 150 with a diamond-tipped stylus. For thickness measurements, a section of a polymer film was removed on each substrate with tweezers to expose the bare silicon surface, and the film thickness was measured as the vertical distance between the surface of the film and the bare substrate. Roughness was measured separately across an unmodified section of the polymer film as the mean deviation of the roughness profile. Experimental trials were run in the hills and valleys mode with a stylus force of 6 mg, a scan length of 2 mm, and a measurement window of 524 μm.

Nuclear magnetic resonance (NMR) 1D spectra were recorded on a 400.13 MHz Bruker AV console equipped with a 9.3 T Oxford Magnet and a 5 mm pulse field gradient broad band probe. Experimental parameters included 16 scans, a recycle delay time of 1.5 s, a 13 ppm sweep width, and 32,000 data points. Multiplicity-edited heteronuclear single quantum coherence (HSQC) experiments were recorded with a 1024 × 256 data matrix, a recycle delay time of 1.5 s, 4 scans per increment, ^13^C GARP decoupling during acquisition, a 34 s multiplicity selection delay, and a *J*(C–H) value of 145 Hz. Data are shown with =CH– and >CH– signals phased positive and −CH_2_– signals phased negative based on π/2 shifted squared sine window function processing. Heteronuclear multiple bond coherence (HMBC) experiments were acquired using a 2048 × 256 data matrix, a *J*(C–H) value of 9 Hz for detection of long-range couplings (resulting in an evolution delay of 55 ms), *J*_1_(C–H) filter delay of 145 Hz (34 ms) for the suppression of one-bond couplings, a recycle delay of 1.5 s, and 128 scans per increment. The HMBC data was processed using a π/2 shifted squared sine window function and displayed in magnitude mode.

Differential scanning calorimetry (DSC) was performed on a TA Instruments Q200 DSC with a TA Instruments Refrigerated Cooling System 90. Samples were removed directly from the silicon substrate using a razor blade and placed in TA Instruments Tzero aluminum pans. Samples were heated to 200 °C, cooled from 200 to −90 °C, and then heated from −90 to 200 °C at a rate of 10 °C/min. The reported traces are from the second heating cycle for each sample.

Attenuated total reflectance Fourier transform infrared spectroscopy (ATR-IR) spectra were obtained using a ThermoFisher Nicolet 6700 FTIR spectrometer equipped with a liquid-nitrogen-cooled mercury–cadmium–telluride detector. Substrates were fastened in the Smart iTR attachment with a diamond crystal plate, and spectra were obtained in the 4000–400 cm^–1^ range.

Contact angles were measured with water, even numbered *n*-alkanes (6 ≤ *n* ≤ 16), diiodomethane, glycerol, and ethylene glycol as the probe liquids using a Ramé-Hart manual contact angle goniometer. Advancing contact angle values were obtained with droplet sizes of ∼ 5 μL and the needle inside the droplet.

Confocal microscopy images were obtained on a Leica SP8 confocal laser scanning microscope operating at 5% power. An air (10×, 0.32 NA) immersion objective was used with a 1 Airy unit pinhole and an 11.2 mm working distance. Films were excited at a 488 nm wavelength, and emission readings were gathered from the 510–550 nm wavelength range. Fluorescence intensities were measured by the green pixel counts in GNU Image Manipulation Program (GIMP) software.

## Results and Discussion

The scROMP film synthesis method displayed in Scheme 1 can simultaneously synthesize and deposit bilayered polymer films by three sequential dispenses. The first dispense is of Grubbs third generation catalyst solution, which prepares the substrate for polymerization upon contact with a cyclic olefin monomer. The second dispense carries the monomer used for the structural bottom layer, which rapidly polymerizes into a film through the combination of polymer growth upward from the substrate with monomer and short-chain oligomer spin-off.^3^ The third dispense carries the second monomer, which polymerizes on top of the first layer to generate a parallel-layered film and thereby governs the surface properties of the assembly. Repeat unit structures of all layered films examined in this study are included in Figure S2 for reference.

### Layered Film Structure

SEM-EDS was chosen as an imaging technique to probe the cross-sectional structures of layered films synthesized by scROMP. Poly­(cyclooctene) (pCOE) was synthesized on top of a poly­(5-(*N*-methyltrichloroacetamide)­norbornene) (pNBMTA) bottom layer to provide a distinctive chlorine signal for the bottom layer in addition to the carbon signal present for both layers. A cross-sectional SEM image of pNBMTA + pCOE is displayed in Figure 1 with elemental maps for silicon, carbon, and chlorine and dashed lines to indicate the substrate (bottom), pNBMTA bottom layer, and pCOE top layer. SEM-EDS images and maps for pCOE and pNBMTA single-layer films are included in Figures S3–S5.

**Figure 1:**
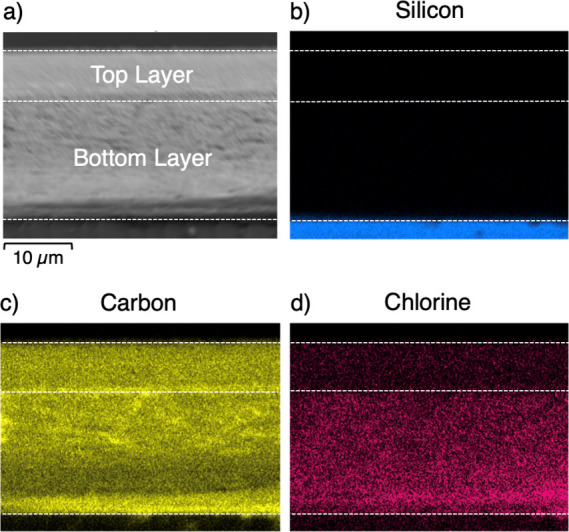
(a) SEM-EDS image of a cross section of a pNBMTA + pCOE film. Elemental maps for (b) silicon, (c) carbon, and (d) chlorine for the same cross section as the first image. Dotted lines are intended to serve as guides to the eye to distinguish between the polymer layers and the substrate.

The carbon map in Figure 1c shows that the layered film is ∼25 μm thick, but the chlorine map in Figure 1d shows a strong signal for only the lower 20 μm. Beyond the 20 μm designation, the chlorine signal drops off precipitously to a level that is comparable to the noise of the chlorine signal in the silicon substrate at the bottom of the map. These maps demonstrate that when monomer is added to growing polymer chains in scROMP, the added monomer forms a distinct outer layer of functionality parallel to the substrate.

### Properties of Polymers from Layered Films

A single initial dispense of the catalyst accounts for the polymerization of both the first and second polymer layers in bilayer films synthesized by scROMP. To assess if the second layer is an outer block of the first layer, gel permeation chromatography and stylus profilometry were combined in Table 1 to investigate whether a second monomer dispense increases the polymer molecular weight and thickness relative to a homopolymer synthesized using a single dispense.

**Table 1:** Number-Average Polymer Molecular Weights (M_N_), Weight-Average Polymer Molecular Weights (M_W_), Dispersities (Đ), and Profilometric Thicknesses of a pNB Homopolymer, a pNB Homopolymer Followed with a Solvent Rinse of Acetone after 60 s, and a pNB + pNB Bilayered Polymer Where the Initial NB Monomer Dispense is Followed by a Second Dispense 60 s Later^a^

film	M_N_ (kDa)	M_W_ (kDa)	dispersity (Đ)	thickness (μm)
pNB	475 ± 12	533 ± 13	1.121 ± 0.001	8.2 ± 0.7
pNB then acetone	500 ± 6	554 ± 6	1.109 ± 0.003	8.0 ± 0.6
pNB + pNB	507 ± 8	571 ± 5	1.127 ± 0.008	9.7 ± 0.7

a Error bars represent ± one standard deviation from the mean from separate injections.

GPC analysis was performed on pNB homopolymer and pNB + pNB bilayered systems to simplify analysis and mitigate solubility issues and viscous fingering of other polymers in this study in common GPC solvents. A pNB film synthesized using a single dispense is demonstrated here to possess high molecular weight (M_W_ ≈ 533 kDa) and low dispersity of 1.121 after 60 s of spin coating. This dispersity is similar to that of 1.13–1.17 we reported for pNBDAC homopolymers^3^ also used herein. When pNB is rinsed with acetone to wash away any unreacted monomer or short-chained oligomers, molecular weight increases slightly, dispersity decreases slightly, and film thickness remains constant, as is expected from the removal of lower molecular weight species by the rinsate. When the acetone rinse is replaced with a second NB dispense, a further increase in molecular weight, a slight broadening of dispersity, and a substantial increase in film thickness are observed. To support that dispersity does not increase significantly in these layered films, Table S1 shows data from GPC for a pNB + pNBDAC film in which dispersity actually decreases after the second layer is formed.

The modest increase in average molecular weight but significant increase in thickness after the addition of the second NB dispense shown in Table 1 precludes a mechanism in which the second dispense produces a second layer that is exclusively a second block of the first layer. Further, given the ∼25 nm radius of gyration for a 500 kDa pNB chain,^45,46^ the notion that chains could initiate near the substrate and stretch all the way to the outer surface of the second layer, several microns away, is incorrect. Instead, unreacted catalyst and shorter-chain oligomers are likely pushed toward the monomer/polymer interface of the growing film due to their increased mobility versus longer, growing chains (the time for a G3 molecule to diffuse across a monomer film was estimated to be ∼4 s in SI.6). These catalysts will continue to initiate new polymer chains throughout the thickness of the film, and the shorter chains will continue to grow, potentially including some diblock and random copolymers near the interface between the layers as the second monomer is added. As thicknesses of the second layers are also well beyond the radius of gyration of polymer chains, new homopolymers must be initiated and propagated as the second layer grows due to the continued presence of shorter chains and unreacted catalyst near the monomer-polymer interface. In this manner, a supply of catalyst and lower molecular weight species is present near the monomer-polymer interface for chain propagation, enabling a remarkably low dispersity for a film grown under 3 min in ambient conditions. The model of this layered film is shown in Figure 2, consisting of a bottom layer that is predominately homopolymer A, a thin region near the interface in which some A-B diblock and/or random copolymers likely exist, and a top layer rich in homopolymer B.

**Figure 2:**
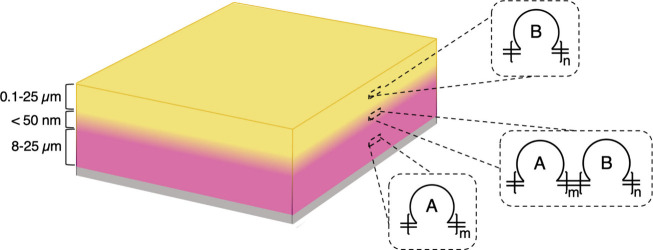
Schematic representation of a cross-section of a layered film synthesized using scROMP with proposed polymer structures and thicknesses of each layer.

Photographs showing the film stability of one-, two-, and three-monomer dispense systems after placement in a solvent that causes delamination of certain film compositions are included in Figure S6 as support for a thin region of copolymers that stabilizes these layered films. In this case, pNBDAC is modified with lysine to generate a water-swellable hydrophilic film. When pNBDAC is synthesized as a homopolymer using one monomer dispense and subsequently placed in the aqueous lysine solution for modification, the film completely delaminates from the substrate and curls significantly. However, depositing the pNBDAC layer atop a robust layer or layers in the two- and three-monomer dispense systems leads to minimal or no delamination or curling, respectively, during modification in an aqueous lysine solution. This enhanced stability of the bi- and trilayered films suggests a strong connection between layers that would be expected if the layers are partially covalently bound to each other to form connective interlayers as opposed to entirely distinct, unconnected polymer layers.

To further investigate polymer structure in pNB + pCOE layered films, 2D NMR was employed to detect the presence of any potential linkages between repeat units from different monomer dispenses on a given polymer chain. Our previous work demonstrated that random copolymer films can be synthesized using scROMP by mixing two comonomers together and spin-coating the monomer mixture through a single monomer dispense.^33^ To compare the polymer structures synthesized here as bilayered films versus those of a single-layered random copolymer, films composed of norbornene and cyclooctene repeat units were synthesized as a random copolymer from one mixed monomer dispense (pNB-r-pCOE) and as a bilayer polymer from consecutive dispenses 60 s apart (pNB + pCOE). The films were then peeled off from their substrates and dissolved in deuterated chloroform for characterization by NMR (Figure 3).

**Figure 3:**
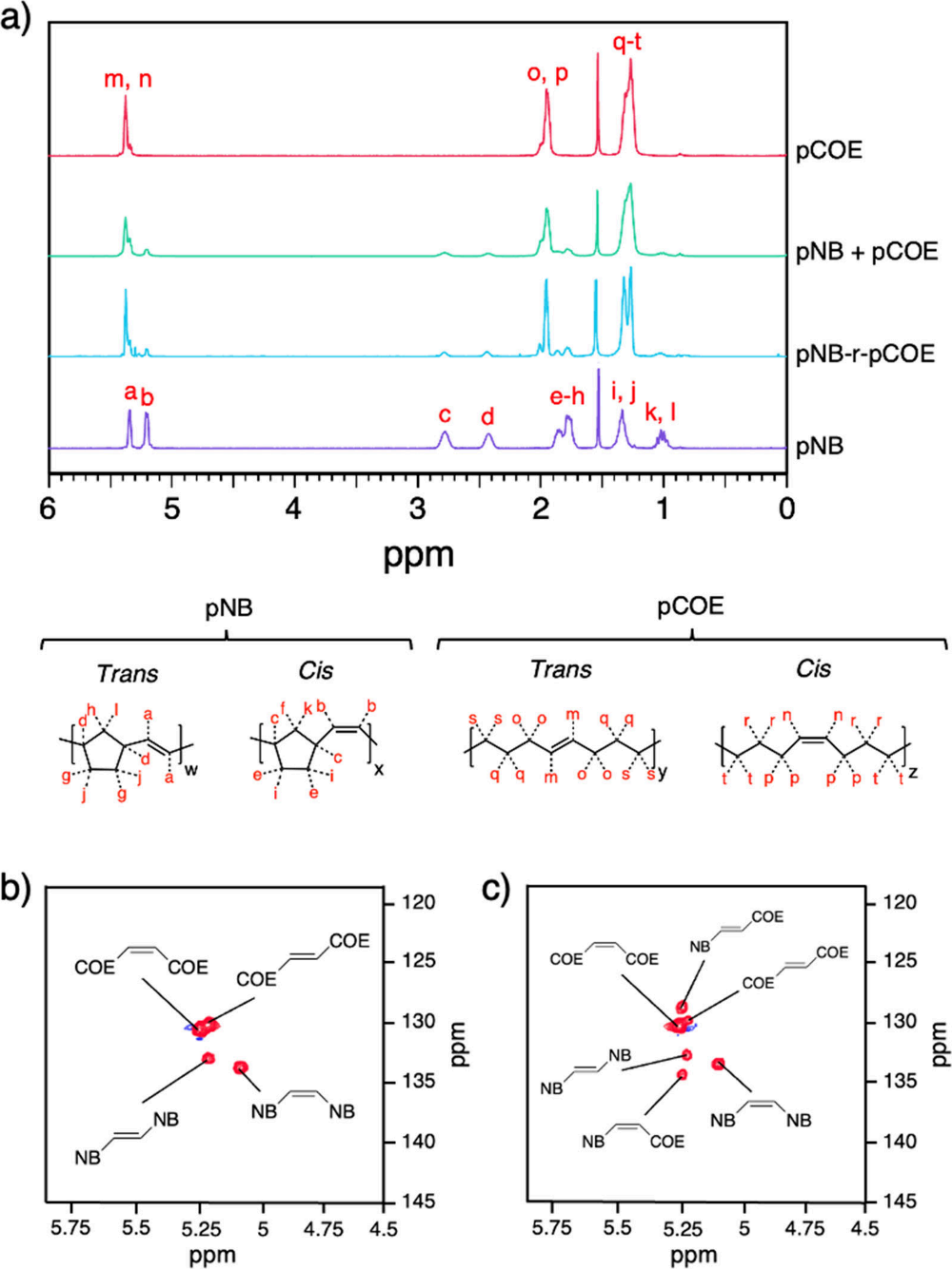
(a) ^1^H NMR spectra of pNB, pNB-r-pCOE, pNB + pCOE, and pCOE films with homopolymer structures and proton labels. HSQC spectra for the olefinic region of (b) pNB + pCOE and (c) pNB-r-pCOE with labels denoting the specific repeat units influencing the signal locations. Full HSQC spectra for pNB + pCOE and pNB-r-pCOE are shown in Figure S7. The sharp peak at 1.55 pm in the 1D spectra is due to trace moisture in the chloroform-d.

NMR spectra for pNB and pCOE homopolymers agree with previously reported NMR spectra for these polymers.^47,48^ The olefinic protons (a, b, m, and n) in pNB and pCOE are far more deshielded than the other protons within the polymers, confirming that unsaturation is maintained in the polymer backbones. The cyclopentyl ring in pNB deshields the protons in the α-position to the olefin (c and d) to a greater extent than the more linear pCOE (o and p) structure does. Chemical shift values for the remaining methylene and methine protons are distinguished based on the proximity to the olefin in pCOE, and both on proximity to the olefin and isomerization of the double bond in pNB.

The ^1^H NMR spectra between the pNB-r-pCOE and pNB + pCOE polymer films are compositionally similar and show characteristic peaks of both pNB and pCOE, albeit at different ratios of pNB to pCOE. We anticipated some copolymerization between NB and COE monomers in the pNB + pCOE film after dispensing the COE monomer, yet HSQC spectra of the alkene region in Figures 3b and 3c and HMBC spectra in Figures S7e and S7f indicate that the degree of copolymerization is sufficiently small to be undetectable. Two additional signals appear in the HSQC spectrum for pNB-r-pCOE in Figure 3c compared to the pNB + pCOE spectrum in Figure 3b, which are assigned based on HMBC spectra in Figure S7 to the presence of olefinic groups within the polymer that are attached to different repeat units on each side of the alkene. Since NB and COE are being simultaneously polymerized in the pNB-r-pCOE film, both repeat units are anticipated to be well-distributed and in close proximity to each other throughout the film. The bilayer polymer spectrum does not show NB-COE attachment and instead shows signals only for olefinic attachment to the same repeat unit, which we attribute to the extremely low concentration of NB-COE connections compared to NB-NB and COE-COE connections in the layered film. A sensitivity analysis based on the degree of polymerization of pNB and the detection limit of ^1^H–^13^C HSQC in SI.9 indicates that even if the entire film consisted of block copolymers (one NB-COE linkage per chain), NB-COE connections would be below common limits of detection for ^1^H–^13^C HSQC. Furthermore, these results confirm that consecutive dispenses do not lead to measurable mixing of the two monomers to form thick regions of random copolymers. Thus, the HSQC spectra agree with the model in Figure 2, suggesting a predominately homopolymer layered structure; the existence of an interlayer of mixed or blocked pNB and pCOE propagation is probable but extremely thin compared to those of isolated pNB and pCOE in the film. Full HSQC spectra for pNB, pCOE, pNB + pCOE, and pNB-r-pCOE are shown in Figures S7a–S7d for reference, and HMBC spectra are shown in Figures S7e and S7f to further support the predominately unconnected structure of chains within that film.

Further characterization confirms that the two monomer-dispense bilayer systems show the collective properties of their constituent homopolymers, while the one monomer-dispense random copolymers show closer to a hybrid of the properties of the constituent homopolymers. Differential scanning calorimetry was performed as shown in Figure 4 on the same polymer films reported in Figure 3 to assess the effect of repeat unit sequencing on the thermal properties of the multicomponent films.

**Figure 4:**
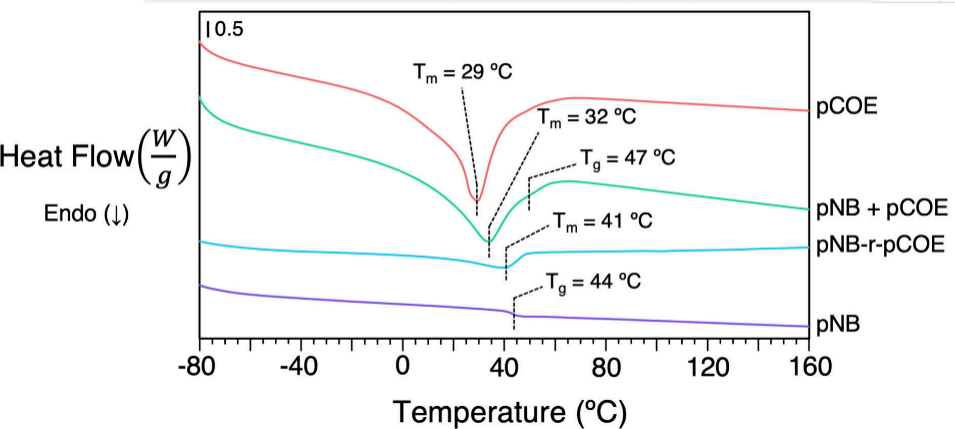
DSC traces from pNB, pNB-r-pCOE, pNB + pCOE, and pCOE films. Traces are from the second heating cycle to erase thermal history.

pNB synthesized by scROMP was previously shown to be a glass at room temperature and did not make the transition to a rubbery state until a temperature of 44 °C.^32^ pCOE does not display a glass transition temperature in the temperature range tested but instead exhibits a melting peak at 29 °C. The greater crystallinity of pCOE versus pNB is attributed to a lack of bulky substituents along the linear pCOE polymer backbone. The pNB + pCOE film demonstrates thermal transitions similar to its homopolymers with a *T*_m_ at 32 °C and an apparent *T*_g_ at 47 °C, indicating that the thermal properties of the individual layers are preserved in these layered films. Such behavior is not observed in the single monomer-dispense pNB-r-pCOE film, which instead displays only one thermal transition—a less well-defined melting temperature that is slightly elevated from the pCOE homopolymer.

### Control Over Wettability and Surface Energy

The existence of a distinct outer layer of functionality in layered films synthesized by scROMP was further demonstrated using wetting measurements, which probe the composition of the outer <0.5 nm of the film.^2^ Contact angle measurements using water and hexadecane as probe liquids are presented in Table 2 for functional top layer compositions on inexpensive lower layer compositions of pDCPD and pCOE. Top layer compositions include modified poly­(norbornene di-acyl chloride) (pNBDAC), whereby two reactive acyl chloride groups per norbornene repeat can be modified with hydrophilic amines or alcohols, pCOE (polymerized on top of pDCPD), and poly­(5-(perfluoro-*n*-alkyl)­norbornene)­s, which are polynorbornenes possessing perfluoro side chain lengths of *n* carbons attached to each cyclopentyl ring. ATR-IR spectra confirm successful polymerization of these layered films (Figures S8 and S9).

**Table 2:** Advancing Contact Angles for Various Top Layer Compositions Using Water (H_2_O) and Hexadecane (HD) as Probe Liquids, and Dispersive (γ_S_^D^), Polar, (γ_S_^P^), and Total (γ_S_) Surface Energy Values Obtained from Zisman and Owens–Wendt Analyses^a^

top layer	θ_A_ H_2_O (°)	θ_A_ HD (°)	$\gamma_{S}^{D}\;\left(\frac{mJ}{m^{2}}\right)$	$\gamma_{S}^{P}\;\left(\frac{mJ}{m^{2}}\right)$	$\gamma_{S}\;\left(\frac{mJ}{m^{2}}\right)$
lysine-modified pNBDAC	22 ± 6	<10	48 ± 1	12 ± 2	60 ± 3
arginine-modified pNBDAC	33 ± 4	<10	48 ± 1	11 ± 7	59 ± 8
hydroxylamine-modified pNBDAC	41 ± 3	<10	45 ± 1	13 ± 3	58 ± 4
pCOE	99 ± 3	34 ± 4	37 ± 1	1 ± 1	38 ± 2
pNBF4	109 ± 4	52 ± 1	17 ± 1	–	17 ± 1
pNBF6	116 ± 2	66 ± 3	13 ± 1	–	13 ± 1
pNBF8	122 ± 2	72 ± 5	7 ± 2	–	7 ± 2

a The modified pNBDAC and pCOE top layers were synthesized on top of pDCPD, and the pNBF*n* top layers were synthesized on top of pCOE.

Lysine-, arginine-, and hydroxylamine-modified pNBDAC top layers all exhibit low contact angles with water as a probe liquid due to the strong polar and hydrogen bonding interactions between their amino, guanidine, carboxylic acid, or hydroxamic acid moieties with water at the solid–liquid interface. Hexadecane wets the surfaces of the three modified pNBDAC surfaces, which is attributed to the low surface tension of hexadecane and the high surface energies of these more polar films.^49^ pCOE and pNBF*n* top layers repel water due to the weaker dispersive interactions between the pCOE or pNBF*n* films and water. pNBF*n* surfaces additionally contain oleophobic fluorocarbon side chains^42^ that repel hexadecane at much larger contact angles (up to 72°) than a fully hydrocarbon pCOE film does (34°).

Wetting measurements can also be used to obtain surface energy estimates for polymer films, which both provide an indication of the functional groups present along the surface of the film and demonstrate the broad applicability of the films. Films with low surface energies are generally effective at repelling probe liquids^49^ and find use as inert coatings,^50^ waterproofing agents,^51^ and solvent-resistant membranes.^52^ Films with higher surface energies cause probe liquids to wet the surface,^49^ minimize fouling,^53^ or enable channel flow in microfluidic devices.^54^ The Zisman^55^ and Owens–Wendt^56^ methods were used in Figures S10 and S11 to obtain estimates for surface energies of the films in Table 2. pNBF*n* top layers exhibit ultralow surface energies of 7, 13, and 17 mJ m^–2^, which is consistent with surface energy values of interfaces composed of a mixture of −CF_3_ (6 mJ m^–2^) and −CF_2_– (18 mJ m^–2^) moieties.^55,57^ pCOE shows an intermediate surface energy value of 38 mJ m^–2^, which is elevated from the surface energy of a polyethylene film lined with only −CH_2_– groups (31 mJ m^–2^)^58^ due to the presence of olefin functional groups along the mostly −CH_2_– polymer backbone. The modified pNBDAC films exhibit polar surface energies that are 11–13 mJ m^–2^ due to the zwitterionic nature of the lysine- and arginine-modified pNBDAC and the strong hydrogen bonding of the hydroxamic acids in the hydroxylamine-modified pNBDAC. The overall (γ_S_) values for these modified pNBDAC top layers are close to the reported values for hydrophilic surfaces within the literature, but exact comparisons between systems are challenging due to the constraints of contact angle-based surface energy estimations for hydrophilic surfaces.^59,60^ Nonetheless, the wetting measurements shown in Table 2 further support the layered structure of films synthesized by two monomer dispenses in scROMP and show that top layers possessing a broad range of hydrophobicities, omniphobicities, and surface energies are attainable via a combined rapid film deposition and synthesis method.

### Control over Top Layer Thickness

pNB + pCOE films on silicon were used as reference systems to assess how top layer thickness varies in response to monomer concentration and spin speed. To assess the effect of monomer concentration on film thickness, COE monomer dispenses atop an 8 μm pNB film were diluted from their neat concentration of 7.7 M down to 0.5 M using pentane, and top layer film thicknesses were measured using stylus profilometry in Figure 5. The effect of COE concentration in the monomer contacting droplet on pCOE thickness when synthesized as a single layer is also shown for reference.

**Figure 5:**
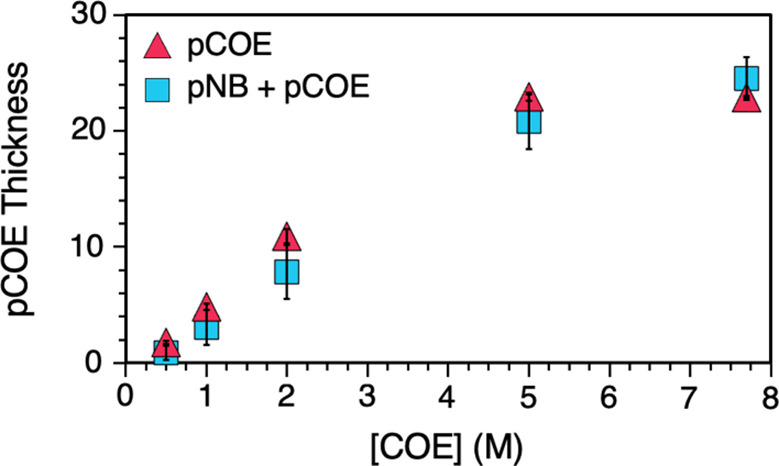
Effect of COE concentration on pCOE thickness as a single layer on a silicon substate (pCOE) and as a top layer on pNB (pNB + pCOE). Spin speed remained constant at 2000 rpm for the catalyst dispense and all monomer dispenses. The bottom layer thickness for pNB in the pNB + pCOE systems was 8 ± 2 μm.

COE concentration in the monomer droplet had a notable effect on thickness in both systems. pCOE formed a thick top layer of ∼25 μm on pNB when synthesized at a neat concentration of 7.7 M, which is distinct from block copolymer films synthesized by surface-initiated methods where reinitiation efficiencies significantly limit outer block thicknesses.^31^ Reducing the concentration in the monomer droplet from 7.7 to 0.5 M decreased thickness by ∼20×, reflecting slower kinetic rates and demonstrating a simple yet effective method to synthesize a thin outer layer of pCOE on pNB. pCOE thickness surprisingly did not statistically deviate between the pCOE single layer and the pCOE top layer in the pNB + pCOE film, implying that a substantial amount of active catalyst must be accessible to COE monomer near the surface of the film after spin coating the first monomer. Modulating spin speed was also explored as a method to thin the outer layer of pCOE in Figure S12; however, changing the spin speed for the COE dispense from 2000 to 6000 rpm while maintaining a 7.7 M concentration did not significantly alter pCOE thickness in the single-layer or two-layer system.

pCOE demonstrates comparable thicknesses when synthesized as a top layer on pNB to when synthesized as a single layer, but many other polymer top layers can be formed on pNB, and thicknesses only sometimes match their single-layer thicknesses. Thicknesses for top layers of pNBDAC, pDCPD, pNBMTA, and pNB on top of pNB are compared to pCOE top layers in Table 3. ATR-IR spectra showing the presence of both layers for the given compositions are indicated in Figure S13.

**Table 3:** Thicknesses of Top Layers of Different Compositions on pNB^a^

composition	thickness when synthesized as a homopolymer (μm)	thickness when synthesized as a top layer on pNB (μm)
pCOE	23 ± 1	25 ± 2
pNBDAC	13 ± 2	9 ± 2
pDCPD	13 ± 3	9 ± 2
pNBMTA	24 ± 4	2 ± 1
pNB	8 ± 2	2 ± 1

a The catalyst dispense and all monomer dispenses were at 2000 rpm. COE and NBDAC were dispensed as neat liquids. DCPD and NBMTA were dissolved and dispensed at 4 M in DCM, respectively. NB was dissolved and dispensed at 2.7 M in pentane.

These results indicate that for pNBMTA and pNB, top-layer thicknesses are not comparable to the thicknesses that are achieved when these compositions are synthesized as single layers. The lower top layer thicknesses of pNBMTA compared with its single-layer thickness could be explained by its larger monomer size, which may impede the ability of NBMTA to sorb into the film and access catalyst molecules from the pNB layer. The lower thickness of the second layer of pNB is difficult to explain from the monomer structure alone because NB is a smaller monomer than either NBDAC or DCPD and is presumably more permeable in the pNB bottom layer than other monomers would be. However, pNB is the only top layer that is synthesized using pentane, which is a significantly worse solvent for pNB than is DCM, and therefore may cause NB monomer from the second dispense to be more easily spun off instead of polymerized and incorporated into the film.

### Three-Layered Systems

Since G3 remains active well after the addition of a first monomer dispense, the use of NB, DCPD, and NBDAC dispenses each 30 s apart from each other was investigated as a model for three-layered films. ATR-IR spectra for pNB, pDCPD, and pNBDAC single-layer films are shown in Figure 6 as references for a three-layered system along with a pNB + pDCPD two-layer film and a pNB + pDCPD + pNBDAC three-layer film before and after modification in an aqueous lysine solution to amidate the pendant acyl chlorides of the pNBDAC layer.

**Figure 6:**
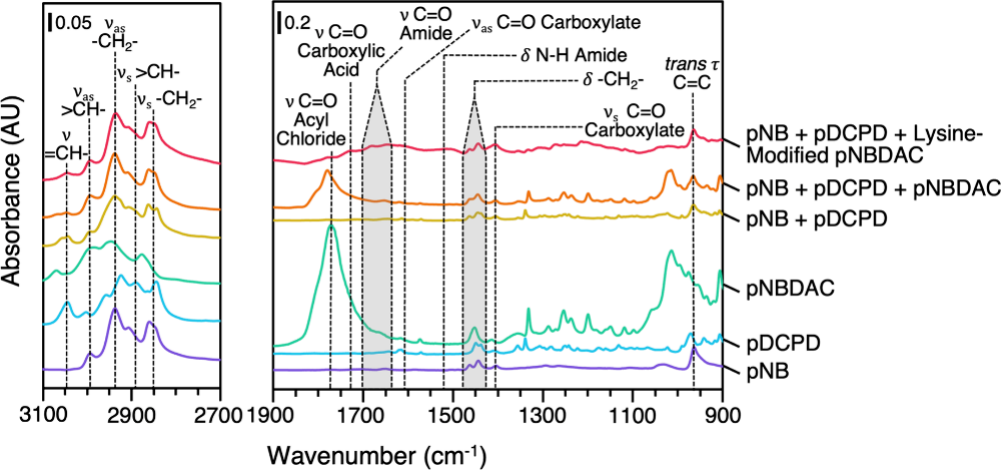
pNB, pDCPD, pNBDAC single-layer; pNB + pDCPD two-layer; and both unmodified and lysine-modified pNB + pDCPD + pNBDAC three-layer spectra. All spectra are normalized so that ν_s/as_ >CH–, =CH–, and −CH_2_– areas are equivalent.

The three single-layer spectra show hydrocarbon stretching (3100–2700 cm^–1^), methylene scissoring (1500–1400 cm^–1^), and olefin out-of-plane bending (∼960 cm^–1^) vibrations that are expected for polymers with unsaturated backbones. pNBDAC shows an additional peak at 1774 cm^–1^ for carbonyl stretching from its dual acyl chloride groups attached to its cyclopentyl rings. The presence of pDCPD, and thus the confirmation of top layer formation in the pNB + pDCPD spectrum, is evident by the enhanced =CH– stretching at 3044 cm^–1^ that is consistent with pDCPD but is not observed in the pNB spectrum. When NBDAC is spun on top of pNB + pDCPD as shown in the pNB + pDCPD + pNBDAC spectrum, a small peak for the ν C=O of the acyl chloride appears, confirming the presence of all three layers in the film. Note that the =CH– stretching beyond 3000 cm^–1^ is diminished relative to the pNB + pDCPD spectrum because ATR-IR only detects the outer few microns of the film,^61^ and a substantial amount of the pNB and pDCPD in the pNB + pDCPD + pNBDAC film is anticipated to be several microns from the film–air interface. The trilayer film was further modified postpolymerization with lysine to generate a surface with distinctly different wetting properties from either of the lower layers. This modification is shown to be nearly complete by the strong diminution of acyl chloride C=O stretching at 1774 cm^–1^ and the formation of the amide C=O stretching and N–H bending at 1646 and 1513 cm^–1^ and carboxylic acid and carboxylate C=O stretching at 1726, 1608, and 1410 cm^–1^. A magnified spectrum for the lysine-modified pNB + pDCPD + pNBDAC is included in Figure S14 to better observe the C=O stretching region associated with the aminolysis reaction.

To confirm that the trilayer system exists in a fully parallel-layered structure, advancing contact angles with water were obtained for the 1-, 2-, and 3-layered adaptations of pNB + pDCPD + pNBDAC and are displayed in Table 4, along with pDCPD and lysine-modified pNBDAC single-layer controls.

**Table 4:** Advancing Water Contact Angles on pNB, pDCPD, Lysine-Modified pNBDAC, pNB + pDCPD, and pNB + pDCPD + Lysine-Modified pNBDAC Films Synthesized on Silicon Substrates

film	θ_A_ H_2_O (°)
pNB	152 ± 2
pDCPD	94 ± 1
lysine-modified pNBDAC	<15
pNB + pDCPD	96 ± 3
pNB + pDCPD + lysine-modified pNBDAC	<15

Contact angles show that the 1-layer pNB film exhibits a particularly high contact angle of 152°. This significantly elevated value from that of many hydrocarbon surfaces is due to the formation of a much rougher film when pentane is used as a solvent. Roughness values from profilometry in Table S2 illustrate the much rougher surface of pNB synthesized using pentane versus when synthesized using a 50/50 v/v% blend of pentane/DCM, the latter of which gives a contact angle with water of only 94°. Pentane was chosen as the solvent for NB for all three-layered systems to best distinguish between the wetting properties of each layer. The 2-layer pNB + pDCPD film shows a drastic drop in contact angle to 96°, maintaining a mildly hydrophobic surface. The 3-layer pNB + pDCPD + lysine-modified pNBDAC shows a drastic change in wetting properties relative to the first and second layers and can be considered superhydrophilic due to a combination of the roughness of the bottom pNB layer and the charged free amino and carboxylate groups present at the outer surface of the three-layered film. Overall, each layer is distinct in its wetting properties from the previous layer and shows wetting properties that are comparable to the wetting properties of the layer when synthesized as a single-layer film.

### Sample Application: Protein-Resistant Surfaces

Biosensors and microfluidic devices require surfaces that minimize the adsorption of proteins for effective use, and careful control over interfacial interactions can greatly reduce the magnitude of undesired adsorption to a device.^62^ Effective antibiofouling has been correlated to the formation of a boundary hydration layer achieved in neutral, hydrophilic coatings that contain hydrogen bond acceptors and do not contain hydrogen bond donors.^63^ Zwitterionic polymers are becoming increasingly popular as replacements for poly­(ethylene glycol) derivatives within the antibiofouling coatings literature as dually charged but neutral coatings exhibit improved long-term stability and more strongly bind the hydration layer using electrostatic interactions instead of hydrogen bonding interactions.^64,65^ Based on pH measurements in Table S3, lysine and arginine are both zwitterionic at 0.1 M in water^66^ and contain deprotonated amine groups that can react with the acyl chloride functional groups on pNBDAC films. Since pNBDAC has stronger reactivity with the neutral, more nucleophilic amine group on lysine or arginine, both lysine- and arginine-modified pNBDAC films should be zwitterionic when modified to form the amide products. scROMP was used as a rapid and simple method to synthesize lysine- and arginine-modified pNB + pDCPD + pNBDAC zwitterionic, three-layered protein-resistant films. Three-layered films were investigated over two-layered systems as the outer pNBDAC layer is more stable against swelling in aqueous protein solutions as shown in Figure S6. pNB, pDCPD, pNB + pDCPD, and lysine-, arginine-, and octanol-modified pNB + pDCPD + pNBDAC films were synthesized and immersed in a solution of fluorescently tagged albumin and imaged using confocal microscopy in Figure 7. Contact angles using water for these surfaces and fluorescence intensity values are included in Table S4, and nonfluorescent reference images are included in Figure S15. An ATR-IR spectrum for octanol-modified pNB + pDCPD + pNBDAC is shown in Figure S16 to verify complete conversion to the octyl ester.

**Figure 7:**
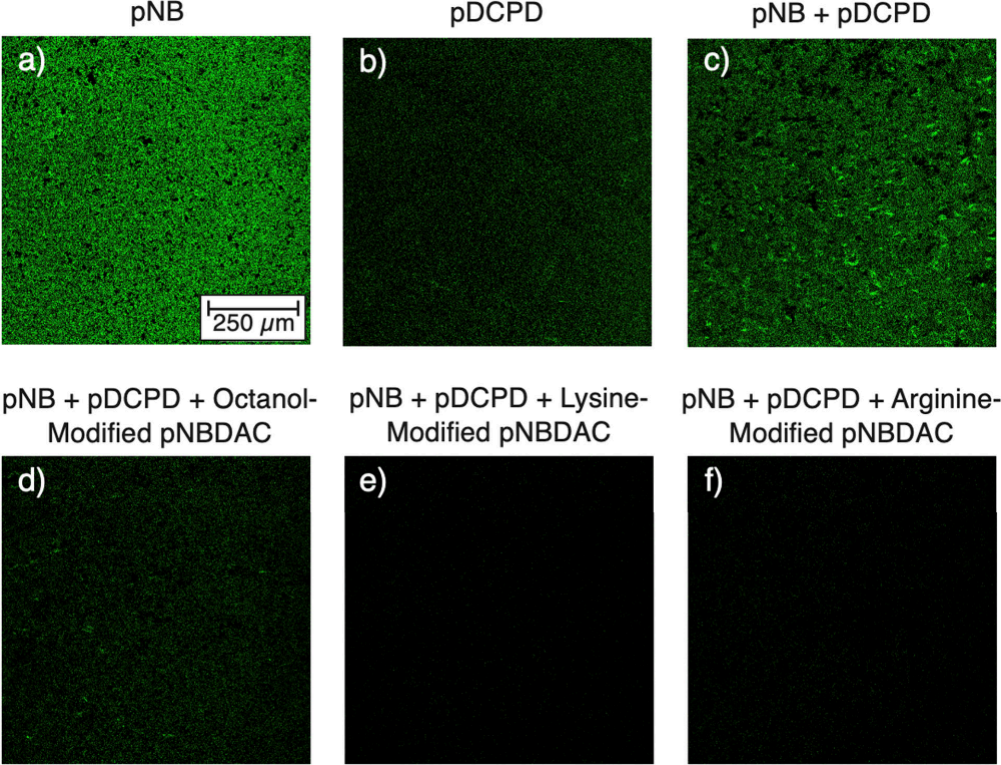
Confocal microscopy images of (a) pNB and (b) pDCPD single-layer films; (c) a pNB + pDCPD two-layer film; and pNB + pDCPD + pNBDAC three-layer films modified with (d) octanol, (e) lysine, and (f) arginine. All films were immersed in a fluorescently tagged albumin solution prior to imaging to assess protein resistance. Films were excited at 488 nm, and emission readings were gathered from 510 to 550 nm.

pNB, being rough and strongly hydrophobic (152°), shows significant albumin adsorption throughout the entirety of the film. The other hydrophobic films, pDCPD, pNB + pDCPD, and pNB + pDCPD + octanol-modified pNBDAC, also show noticeable adsorption but not at the same intensity as pNB, which is in part explained by their ∼60° lower contact angles. Film roughness also appears to play an important role in the protein-resistant properties of the films. Fluorescence intensity shown is only about one-third the intensity in pDCPD than in pNB + pDCPD, and the latter has the same contact angle but possesses a much larger visual roughness based on the nonfluorescent confocal images in Figure S15. Lysine- and arginine-modified pNB + pDCPD + pNBDAC films show significantly reduced albumin adsorption relative to any of the hydrophobic films. Fluorescence intensity is 50× and 25× less for lysine- and arginine-modified three-layer films relative to pNB and 37× and 18× less relative to the pNB + pDCPD, demonstrating the improved antibiofouling properties of those zwitterionic surfaces over hydrophobic surfaces. Here, scROMP provides a versatile strategy to rapidly prepare multilayered films that have inner hydrophobic structural layers and outer zwitterionic surfaces that are resistant to the adsorption of proteins.

## Conclusions

The synthesis of layered films using traditional methods can be time- or step-intensive and often produces films that are difficult to characterize with bulk techniques. The advancement of many industries necessitates the development of specialty coatings and membranes with targeted surface interactions that are frequently expensive or challenging to produce in bulk and therefore best confined to the interface of the film. The scROMP approach here integrates spin coating and polymerization together to minimize solvent usage, time, and the number of steps to fabricate functionalized surfaces on inexpensive but structurally supportive lower layers. scROMP employs the fast and living polymerization kinetics of Grubbs third generation catalyst to add functional polymer to the surface of a growing film, thus maintaining a surface layer of the targeted functionality while controlling the overall incorporation of the pricey monomer within the film. This process is extremely rapid, producing films with polymers of >500 kDa molecular weight and <1.15 dispersities in less than 3 min while using less than 600 μL of solvent per 2.25 cm^2^ film. NMR and DSC data show that these layered films possess similar compositional and thermal properties to their respective homopolymers, and SEM-EDS images visually verify the formation of distinct layers parallel to the substrate. Thicknesses of the top layers can be controlled through monomer concentration, demonstrating top layer thicknesses from extremely thin to three times the thickness of the lower layer.

scROMP was additionally shown here to synthesize top layers with compositions that are relevant to several active areas of thin film research. The layered films in this study show surface energies from 7 to 60 mJ m^–2^ and a wide array of hydrophobicities and omniphobicities. The more hydrophilic top layers greatly minimize protein adsorption relative to their hydrophobic lower layers. The results presented here illustrate the synthetic ease of utilizing scROMP as a polymeric materials discovery method for layered films relative to existing methods. From the perspectives of efficiency and sustainability, scROMP enables the rapid conversion of cyclic olefin monomers to layered films of distinct functionality and is an attractive method for the exploration of future layered materials and coatings.

## Supplementary Material



## Notes

NMR spectrum of a synthesized monomer, polymer structures for all two- and three-layered compositions, HSQC and HMBC spectra for layered polymers and random copolymers, NMR sensitivity analysis, catalyst diffusion time estimate through the film, SEM-EDS homopolymer maps, numerous ATR-IR spectra, a Zisman plot, an Owens–Wendt plot, pH values for select modification solutions, thickness plots as functions of spin speed, roughness values from stylus profilometry, additional contact angles, fluorescence intensities for confocal images, and nonfluorescent confocal images (PDF)
