# Supplementary material for: Rapid Deposition of Layered Polymer Films by Ring-Opening Metathesis Polymerization during Spin Coating
Source: ACS Appl Polym Mater. 2026 Jun 23;8(13):10333–44. doi: 10.1021/acsapm.6c01078 (PMC13366571; doi:10.1021/acsapm.6c01078)
Supplement: Supplementary file 1 [file ap6c01078_si_001.pdf]

Supporting Information

Rapid Deposition of Layered Polymer Films by Ring-Opening Metathesis Polymerization during Spin Coating

Matthew P. Vasuta,[1] Skyler T. Hornback,[2] and G. Kane Jennings[2,]*

[1]Interdisciplinary Materials Science Program, Vanderbilt University, Nashville, Tennessee, 37235, United States.

[2]Department of Chemical and Biomolecular Engineering, Vanderbilt University, Nashville, Tennessee, 37235, United States.

Corresponding Author: *G. Kane Jennings, Department of Chemical and Biomolecular Engineering, Vanderbilt University, Nashville, TN, 37235; Email: kane.g.jennings@vanderbilt.edu

SI.1 – ^{1}H NMR of 5-(*N*-methyltrichloroacetamide)norbornene (NBMTA)

5-(*N*-methyltrichloroacetamide)norbornene was synthesized via dropwise addition of trichloroacetyl chloride into a round-bottom flask of norbornene methylamine ($NBNH_2$) as described in the "Materials and Methods" section of this paper. ^{1}H NMR spectra of the solid NBMTA product and the as received $NBNH_2$ from Tokyo Chemical Industry Co. are shown in Figure S1.

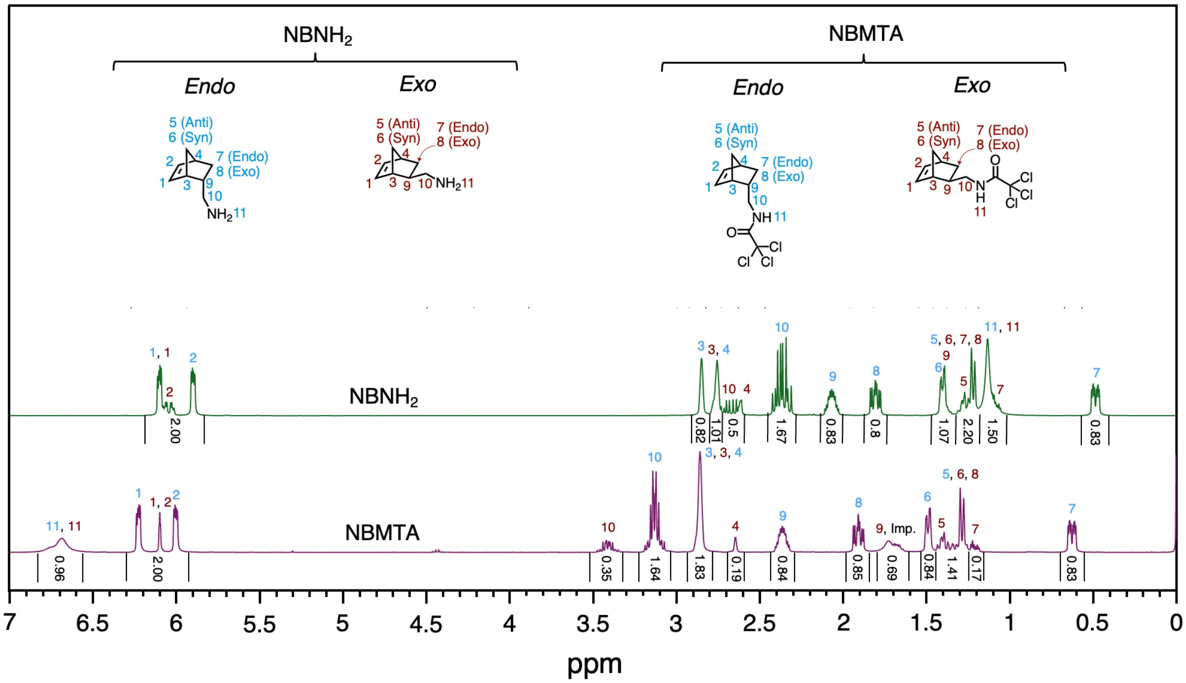

Figure S1. ^{1}H NMR spectra of norbornene methylamine ($NBNH_2$) as received from Tokyo Chemical Industry Co., Ltd. and the synthesized 5-(*N*-methyltrichloroacetamide)norbornene (NBMTA) with their associated structures showing protons of different chemical shifts. Both samples were dissolved in chloroform-d.

The endo/exo ratio in the $NBNH_2$ monomer stock solution is 0.83:0.17, which is maintained in the NBMTA product. Successful modification of the amine to the amide product is demonstrated by the replacement of the amine proton peaks at a chemical shift of 1.2 ppm (labelled as "11") by the peaks at 6.7 ppm, which are within the typical chemical shift range for protons on a secondary

amide.[1] The protons at the α-position to the amide (labelled as "10") are also consistent with the successful formation of the amide product in the NBMTA spectrum.[1]

SI.2 – Layered Polymer Structures

Many polymer compositions are used throughout this study, so reference structures for all polymers used in this study are included in Figure S2. The first layer is denoted with an "x" subscript, the second layer with "y", and the third layer (if part of the structure) is labeled with "z".

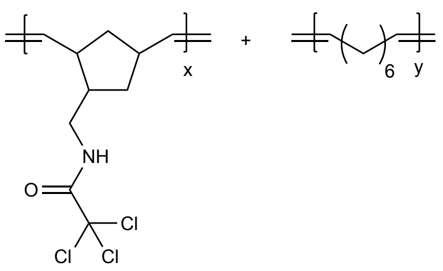

pNBMTA + pCOE
Figure 1

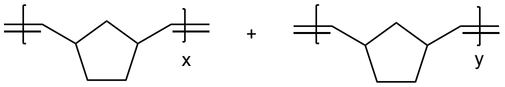

pNB + pNB
Table 1, Table 3, and Figure 2

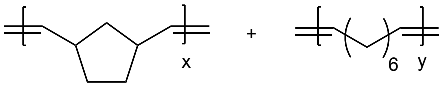

pNB + pCOE
Figure 3, Figure 4, Figure 5, and Table 3

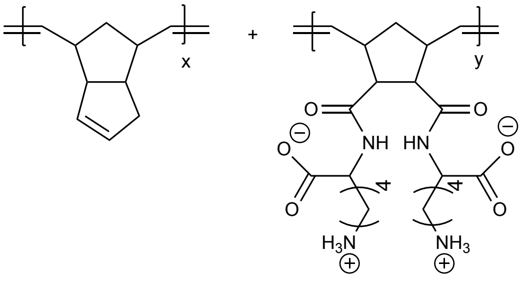

pDCPD + Lysine-Modified pNBDAC
Table 3

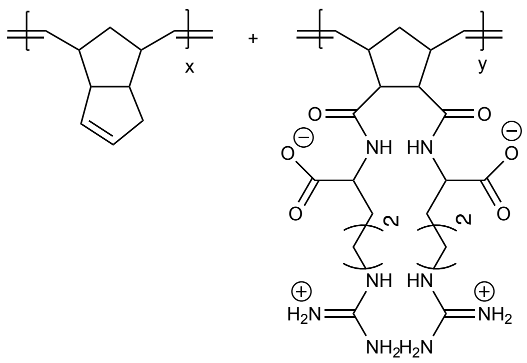

pDCPD + Arginine-Modified pNBDAC
Table 2

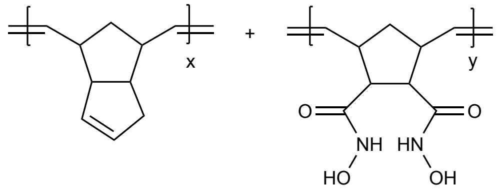

pDCPD + Hydroxylamine-Modified pNBDAC
Table 2

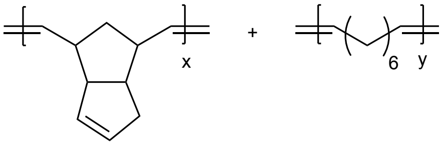

pDCPD + pCOE
Table 2

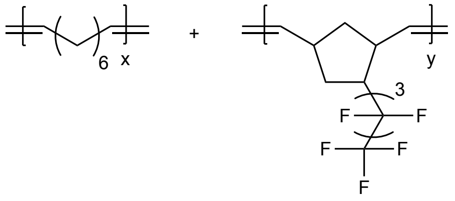

pCOE + pNBF4
Table 2

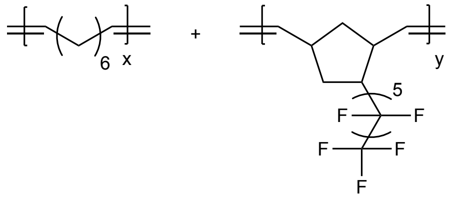

pCOE + pNBF6
Table 2

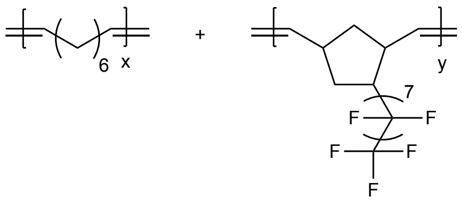

pCOE + pNBF8
Table 2

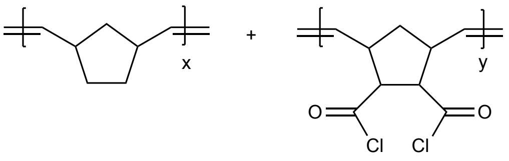

pNB + pNBDAC
Table 3

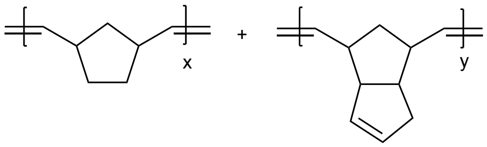

pNB + pDCPD
Table 3, Table 4, Figure 5, and Figure 7

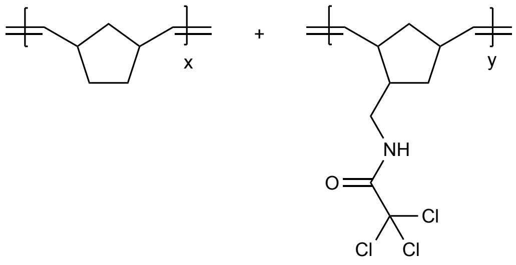

pNB + pNBMTA
Table 3

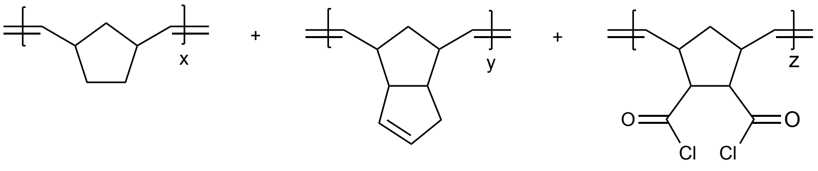

pNB + pDCPD + pNBDAC

Figure 6

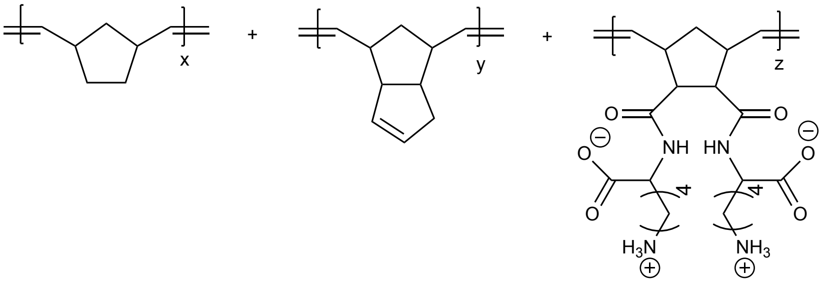

pNB + pDCPD + Lysine-Modified pNBDAC
Figure 6, Figure 7, and Table 4

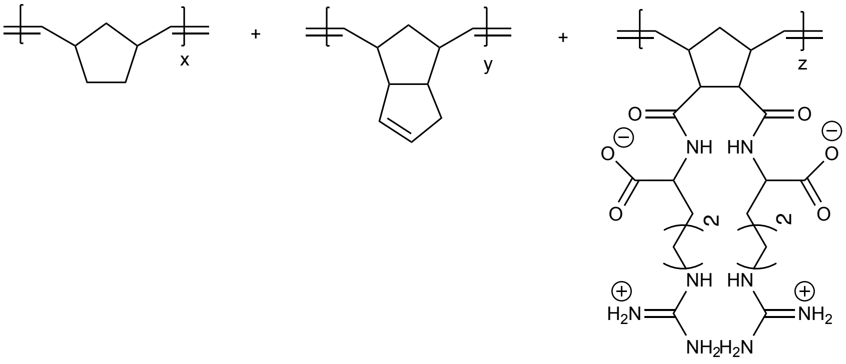

7

pNB + pDCPD + Arginine-Modified pNBDAC
Figure 7

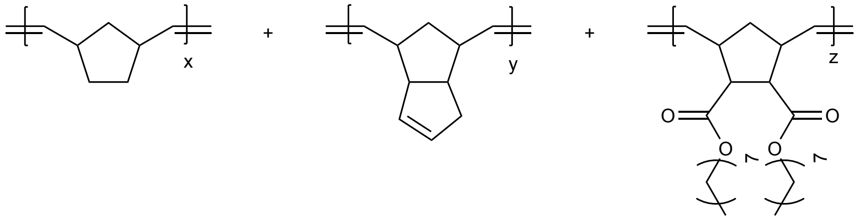

pNB + pDCPD + Octanol-Modified pNBDAC
Figure 7

Figure S2. Polymer structures used throughout this study with the associated figures that the structures appear in.

SI.3 – pCOE and pNBMTA SEM-EDS Homopolymer Images and Maps

SEM-EDS images and elemental maps of pCOE and pNBMTA homopolymers were obtained in Figure S3 and S4, respectively, as references for the expected map signals of the two homopolymers used as separate layers in Figure 1.

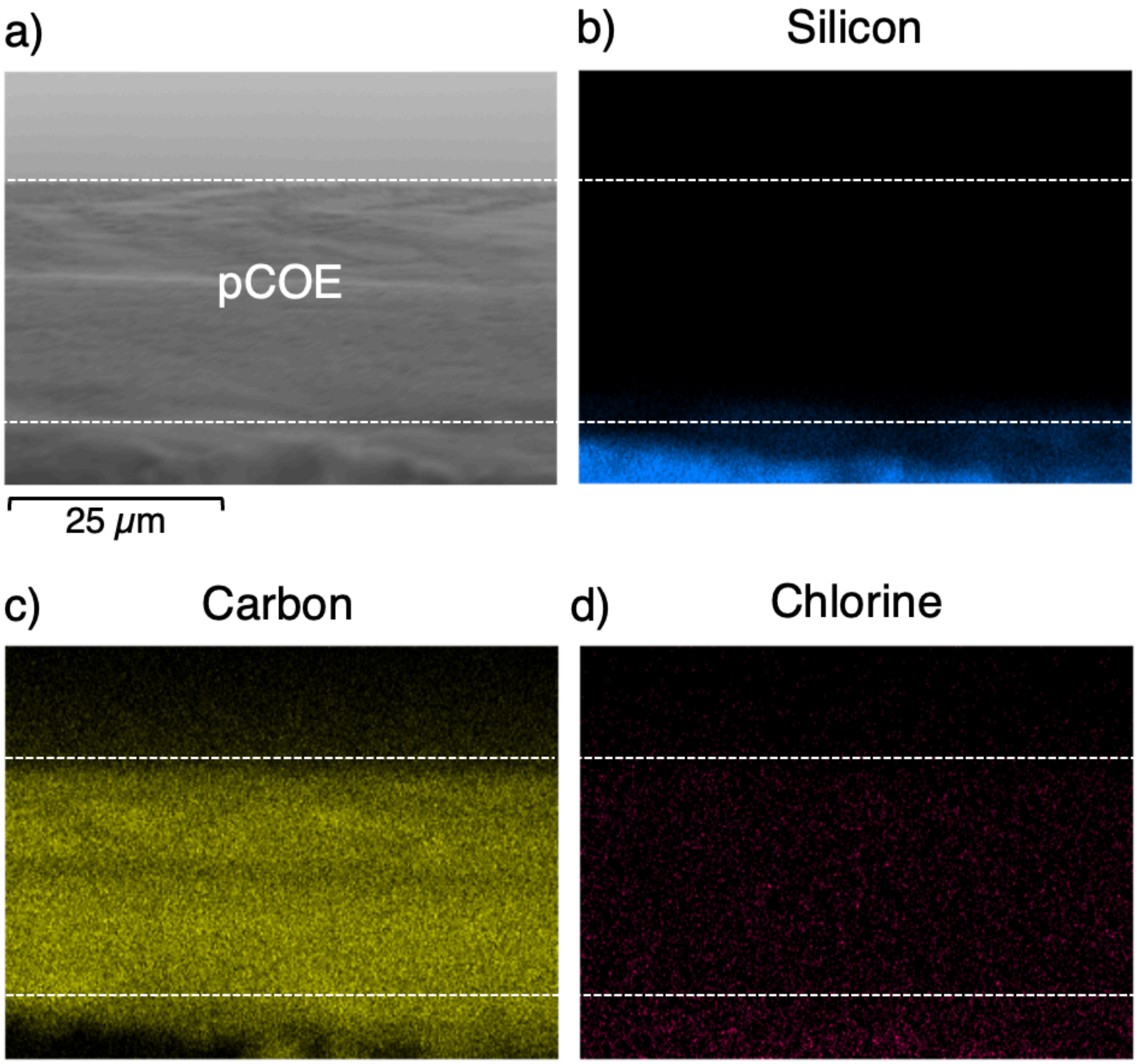

Figure S3. a) SEM-EDS image of a cross-section of a pCOE film. Elemental maps of the pCOE image for b) silicon, c) carbon, and d) chlorine. Dotted lines are intended to serve as guides to the eye to distinguish the film from the substrate.

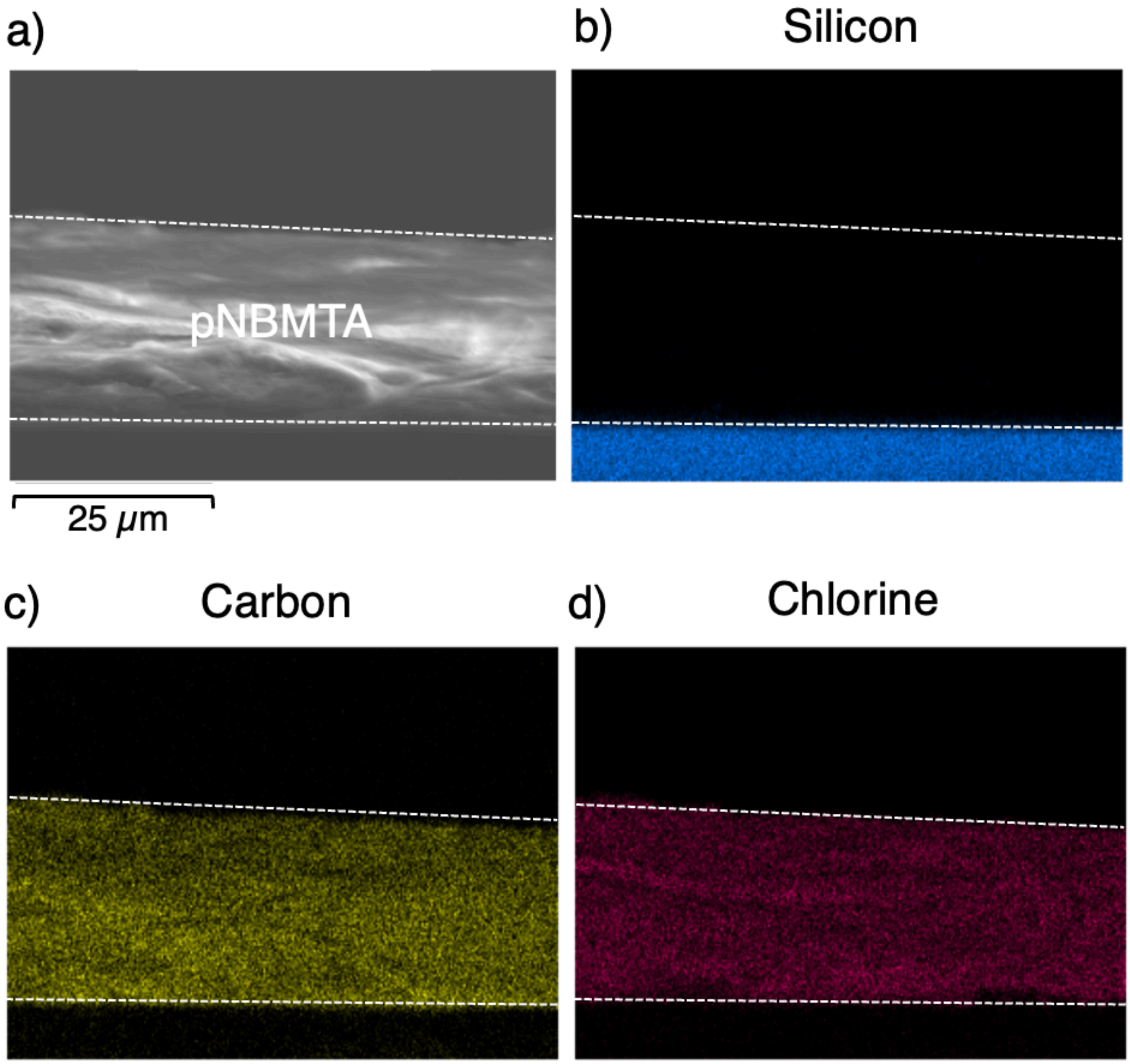

Figure S4. a) SEM-EDS image of a cross-section of a pNBMTA film. Elemental maps of the pNBMTA image for b) silicon, c) carbon, and d) chlorine. Dotted lines are intended to serve as guides to the eye to distinguish the film from the substrate.

The carbon map for the pCOE homopolymer shows an ~25 μm thick polymer film with some degree of overhang across the silicon substrate. The chlorine signal between the polymer film and the substrate are comparable as would be anticipated from the fully hydrocarbon makeup of pCOE, although some amount of chlorine is anticipated to be present in the pCOE chlorine map from chlorine in the Grubbs 3rd generation catalyst. The carbon map for the pNBMTA homopolymer in

Figure S4b also reveals a ~25 µm thick film without a substantial overhang of polymer toward the substrate. The chlorine signal in Figure S4d is strong throughout the entire depth of the film in comparison to the silicon substrate in the same map, consistent with that which was observed with the bottom layer in the pNBMTA + pCOE chlorine map in Figure 1d.

SI.4 – Attempts at Ru Detection within Layered Films

We attempted to identify G3 within films synthesized by scROMP by mapping Ru in Figure S5b for the cross-sectional SEM-EDS image of pCOE in Figure S3.

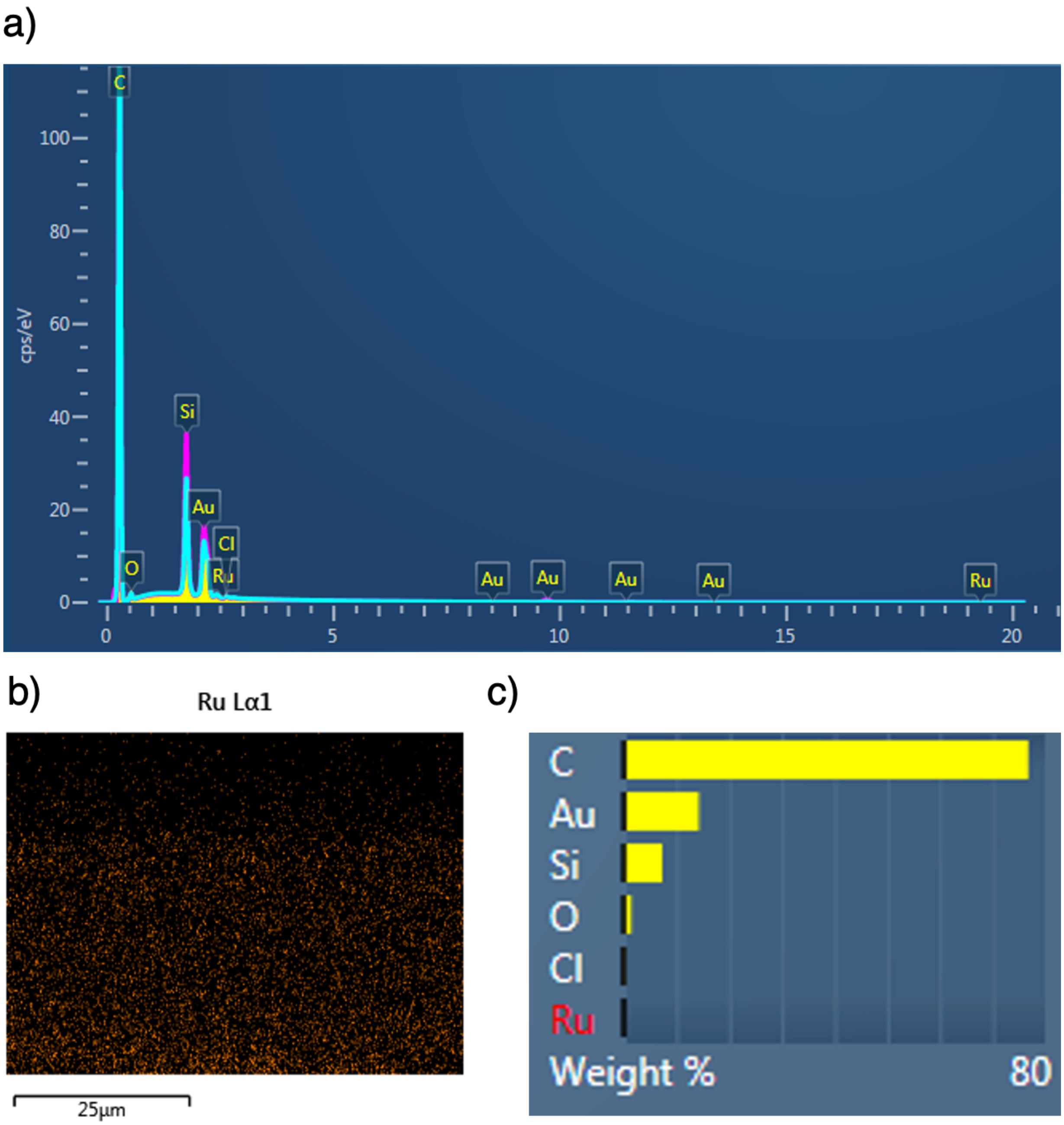

Figure S5. a) EDS spectrum, b) ruthenium map (Lα1), and c) elemental weight percentages (obtained from EDS) for the pCOE SEM-EDS image displayed in Figure S3.

An EDS spectrum in Figure S5a and the elemental weight percentages in Figure S5c show that Ru is not detected in appreciable quantities within the polymer film and suggest that SEM-EDS cannot identify the cross-sectional distribution of G3 within the film. As a result, an unreliable signal for

ruthenium appears throughout the entire system, including in the substrate and in the headspace above the film, but the signal is largely the result of forcing the EDS system to fit Ru rather than the actual confident detection presence of Ru in the film. Assuming one catalyst per polymer chain at an average molecular weight of 500 kDa, the level of Ru in the film would be only 200 ppm and well below the 1000 ppm detection threshold recommended for EDS.[2]

SI.5 – pNB + Ethanol-Modified pNBDAC GPC

GPC and profilometry were performed on a separate pNB + ethanol-modified pNBDAC system in Table S1 from that of pNB + pNB reported in Table 1 to determine if changing the composition of the top layer dramatically altered molecular weight and dispersity trends.

Table S1. Number-average polymer molecular weights (M_N), weight-average polymer molecular weights (M_W), dispersities ($Đ$), and profilometric thicknesses of a pNB homopolymer, a pNBDAC homopolymer, and a pNB + pNBDAC bilayered polymer where the initial NB monomer dispense is followed by an NBDAC dispense 60 s later and then modified post-polymerization with ethanol. Error bars represent +/- one standard deviation from the mean from separate injections. pNB was synthesized with different catalyst and monomer batches, which may account for its lower molecular weights compared to those reported in Table 1. Thickness values for ethanol-modified pNBDAC were obtained before modification to form the ethyl ester.

Film	M_N (kDa)	M_W (kDa)	Đ	Thickness (μm)
pNB	302 ± 11	391 ± 4	1.29 ± 0.03	8 ± 1
Ethanol Modified pNBDAC	299 ± 16	396 ± 14	1.33 ± 0.03	13 ± 2
pNB + Ethanol Modified pNBDAC	341 ± 13	420 ± 18	1.23 ± 0.01	17 ± 3

Similar to the pNB + pNB system, changes in molecular weight and dispersity were relatively small in adding the second layer. The pNB and ethanol-modified pNBDAC homopolymers exhibit similar molecular weights and dispersities to one another and layering the ethanol-modified pNBDAC layer on pNB only mildly increased molecular weight and decreased dispersity of the overall system while increasing film thickness dramatically. These results further confirm that molecular weights and dispersities are relatively unchanged by the addition of the second layer.

SI.6 – Diffusion of G3 in a Monomer Film

The high molecular weights and low dispersities in polymer film growth using scROMP were attributed to the greater mobility of catalyst molecules and low molecular weight species to diffuse across the polymer/monomer gel to maintain propagation via reaction with free monomer.. To ensure the timescales of diffusion are consistent with the polymerization time of the scROMP process, we estimated the diffusion of a catalyst molecule through a monomer film using the Stokes-Einstein[3,4] equation:

$$D = \frac{k_B T}{6\pi\mu r} \quad (S1)$$

where D is the diffusion coefficient, k_B is the Boltzmann constant, T is the temperature, μ is the dynamic viscosity of the fluid, and r is the radius of a spherical particle. Assuming an ~ 2 nm radius for G3 and a cyclooctene viscosity of 1.89 mPa·s, the estimated diffusion coefficient is:

$$D = \frac{\left(1.31 \cdot 10^{-23} \frac{J}{K}\right)(289\ K)}{6\pi(1.89 \cdot 10^{-3}\ Pa \cdot s)(2 \cdot 10^{-9}\ m)} = 5.3\ \cdot 10^{-11}\ \frac{m^2}{s} \quad (S2)$$

The time for G3 to diffuse across a 15 μm film of monomer is then:

$$t = \frac{L^2}{D} = \frac{(15 \cdot 10^{-6}\ m)^2}{5.3 \cdot 10^{-11}\ \frac{m^2}{s}} \approx 4\ s \quad (S3)$$

A diffusion time of 4 s for G3 molecules is consistent the assumption that catalyst molecules can easily diffuse through liquid-like regions of the growing film within the 120 s of polymerization time.

SI.7 – Photographs of 1-, 2-, and 3-Layered Lysine-Modified pNBDAC Films

To demonstrate the effect of layering on the stability of films synthesized by scROMP, photographs were obtained of films after immersion in a 0.1 M lysine$_{(aq)}$ solution when pNBDAC is synthesized as a single layer, as a second layer on top of pDCPD, and as a third layer on top of

pNB and pDCPD (Figure S6). Lysine can modify pNBDAC to form the amide product as shown in Figure S2, and this layer is easily swollen in the aqueous modifying solution.

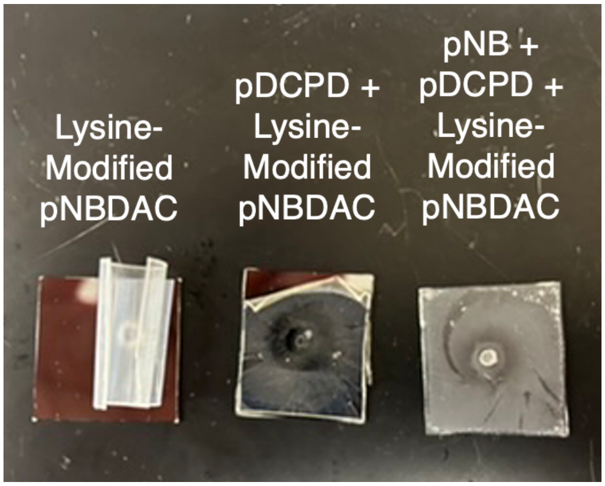

Figure S6. Photographs of lysine-modified pNBDAC as a single layer, as the top layer in a two-layer film with pDCPD as the bottom layer, and as the top layer in a three-layered film with pNB and pDCPD as the bottom and middle layers, respectively.

The single-layer lysine-modified pNBDAC exhibits significant adhesion issues with the substrate because the aqueous lysine solution penetrates the film and lifts the film off of the substrate. Synthesizing the pNBDAC layer on a more hydrophobic pDCPD bottom layer drastically reduces these adhesion issues, and delamination issues are only observed around some of the corners of the film in which the aqueous solution penetrates between the film and substrate. Importantly, the limited delamination observed at the edges is for the entire two-layered film and not just the hydrophilic pNBDAC layer, indicating robust connection between layers. Forming the pNBDAC layer with an additional hydrophobic pNB layer below mitigated adhesion issues altogether, and no delamination of any layers was observed. If the layers formed as independent homopolymers on top of each other, we expect that the pNBDAC layer in the 1-, 2-, and 3-layered film would delaminate from the rest of the polymer film when placed in the lysine solution, as it does as a single-layered homopolymer. These photographs instead show connectedness between layers, as

the lysine-modified pNBDAC layer does not delaminate from the polymer layers when synthesized as a top layer.

SI.8 – HSQC and HMBC Spectra of pNB + pCOE and pNB-r-pCOE Films

Full HSQC spectra for the polymer films in Figure 3 are displayed in Figure S7a-S7d for pNB, pCOE, pNB + pCOE, and pNB-r-pCOE, respectively, along with HMBC spectra for pNB + pCOE at lower ^{13}C chemical shifts (Figure S7e) and higher ^{13}C chemical shifts (Figure S7f).

a)

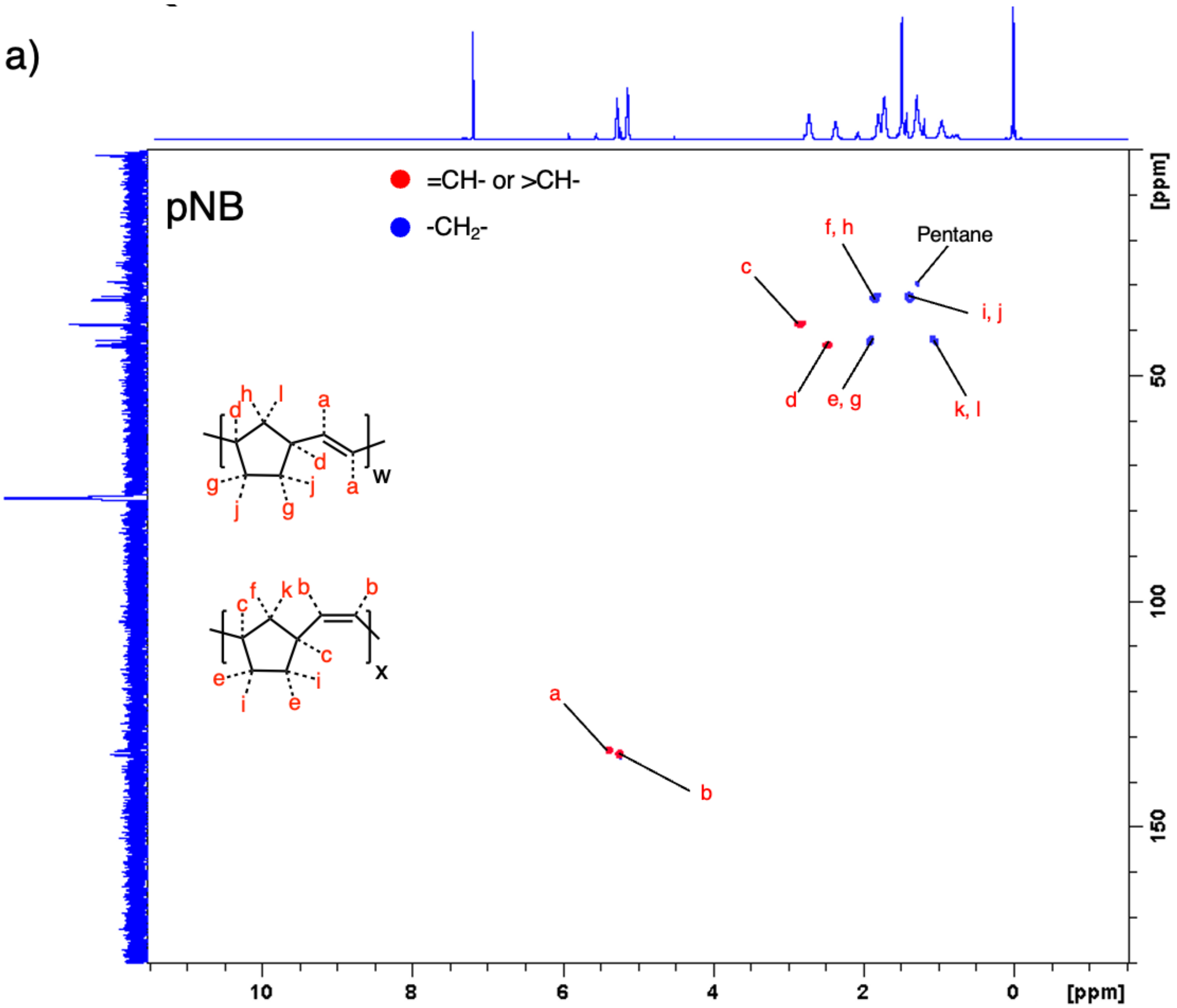
pNB
=CH- or >CH-
-CH2-
f, h
Pentane
c
i, j
d
e, g
k, l
a
b
w
x
10
8
6
4
2
0
[ppm]
50
100
150
[ppm]

b)

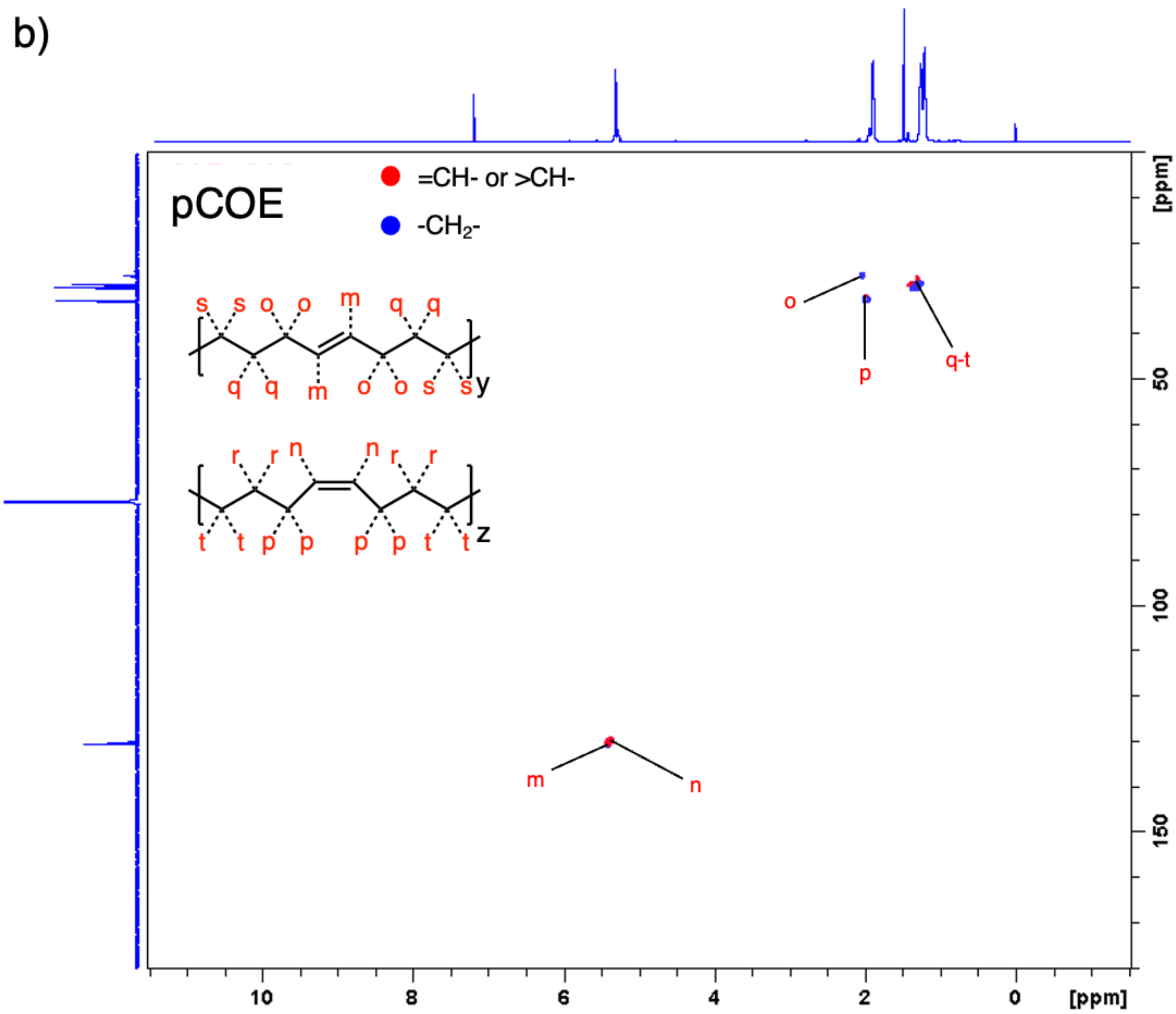

pCOE
=CH- or >CH-
-CH2-
s s o o m q q
q q m o o s s
y
r r n n r r
t t p p p p t t
z
o
p
q-t
m
n
10
8
6
4
2
0
[ppm]
50
100
150
[ppm]

c)

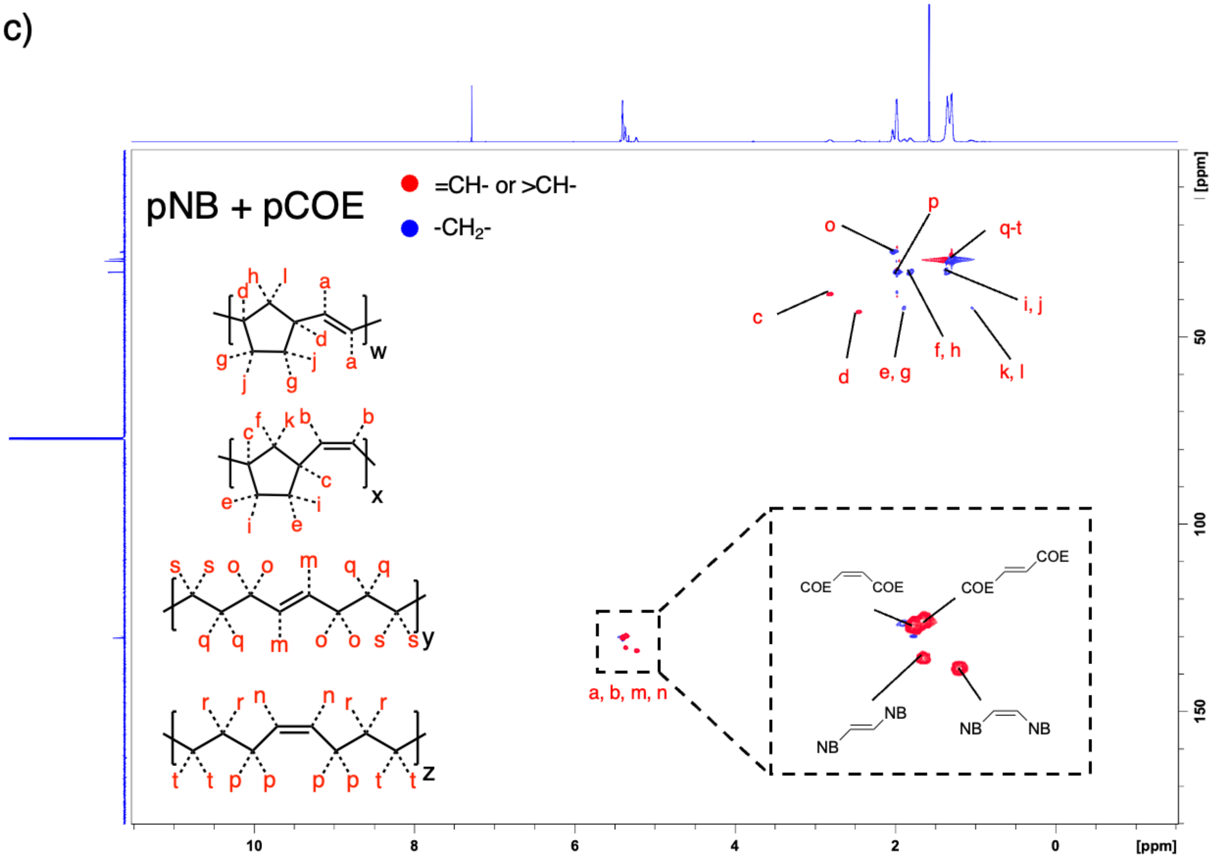

d)

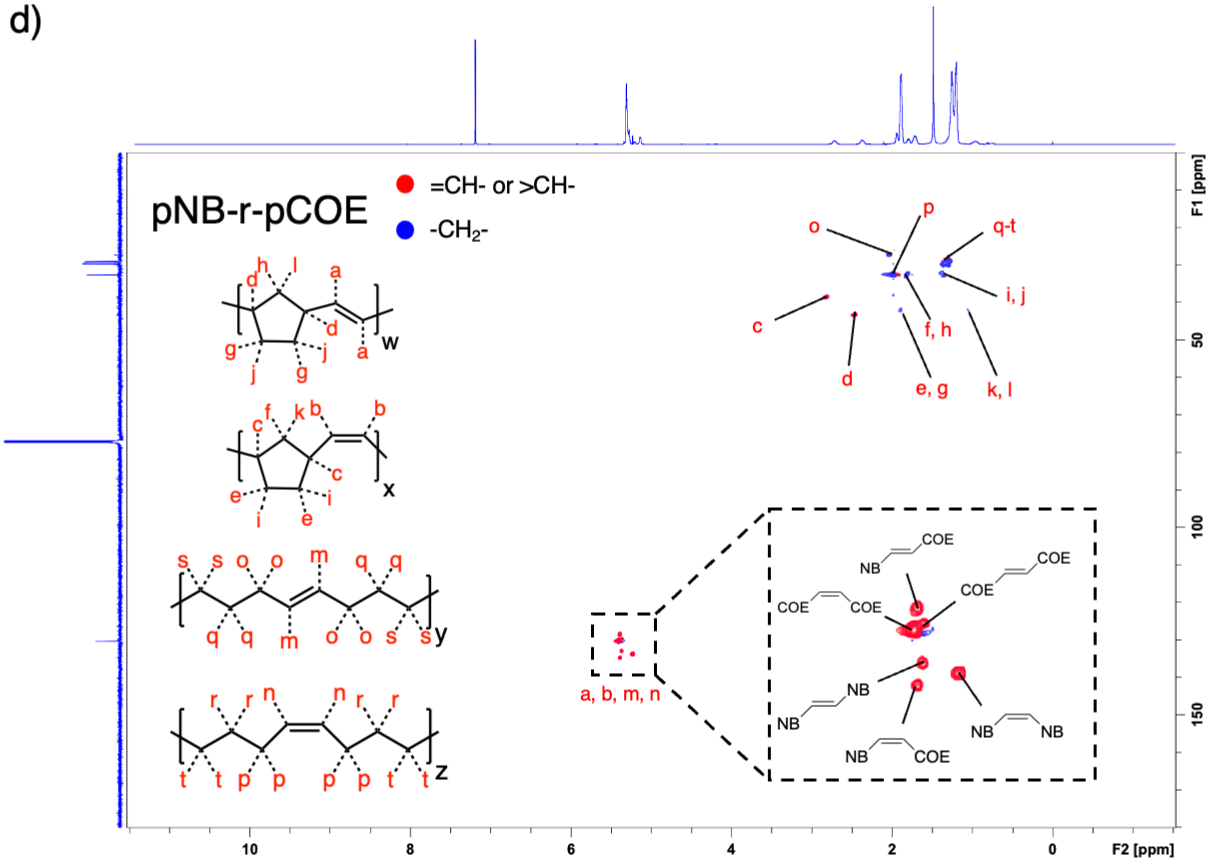

pNB-r-pCOE
=CH- or >CH-
-CH2-
o
p
q-t
i, j
c
f, h
d
e, g
k, l
a, b, m, n
NB COE
COE COE
COE COE
NB NB
NB NB
NB COE
F1 [ppm]
F2 [ppm]

e)

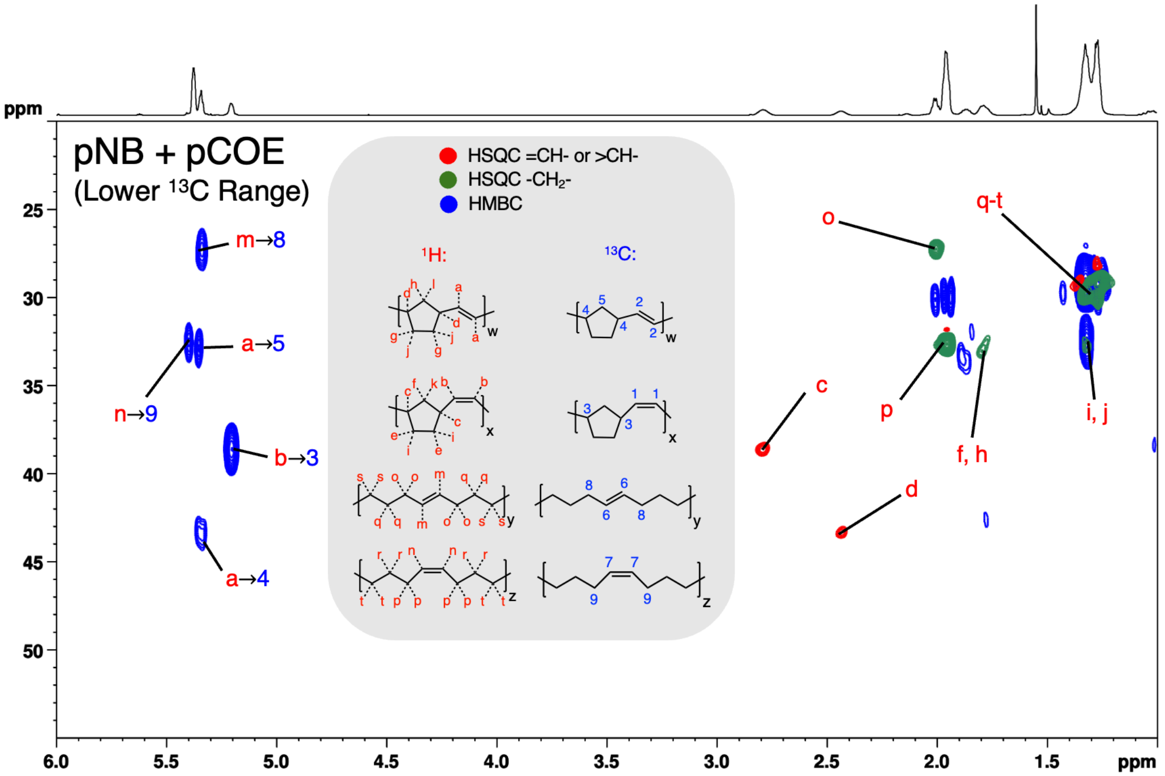

pNB + pCOE
(Lower 13C Range)
HSQC =CH- or >CH-
HSQC -CH2-
HMBC
1H:
13C:
m→8
a→5
n→9
b→3
a→4
o
q-t
c
p
i, j
f, h
d

f)

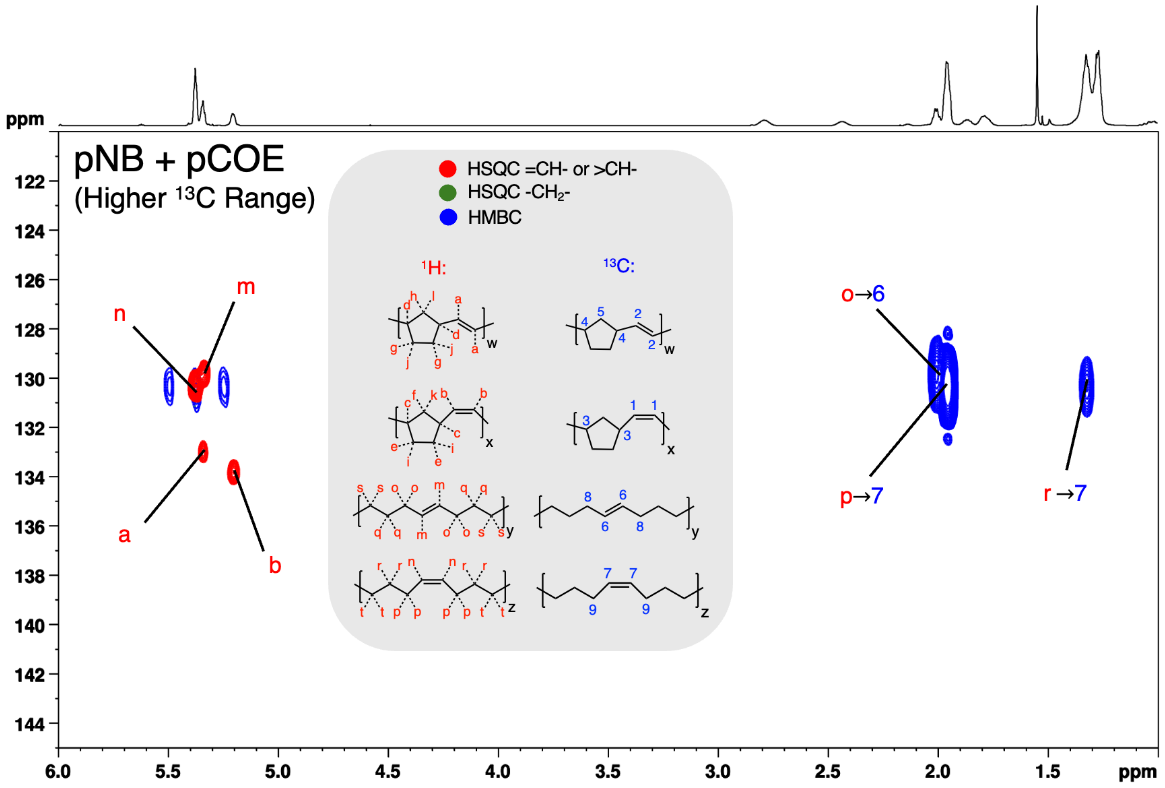

pNB + pCOE
(Higher 13C Range)
HSQC =CH- or >CH-
HSQC -CH2-
HMBC
1H:
13C:
n
m
a
b
o→6
p→7
r→7

Figure S7. HSQC of a) pNB, b) pCOE, c) pNB + pCOE, and d) pNB-r-pCOE with their ^{1}H and ^{13}C spectra projected on the x- and y-axes, respectively. HMBC of pNB + pCOE in the e) less deshielded ^{13}C region and f) more deshielded ^{13}C region with their ^{1}H and ^{13}C spectra projected on the x- and y-axes, respectively. Red labels denote the ^{1}H responsible for the signal, and blue labels (if present) denote the ^{13}C to which the ^{1}H is coupled to.

Since HSQC experiments are primarily concerned with single-bond couplings between ^{1}H and ^{13}C atoms, HMBC experiments were performed on the pNB + pCOE film for explicit identification of longer-range couplings. HMBC spectra for the pNB + pCOE film further confirm that in appreciable concentrations, only NB connects to NB and only COE connects to COE. This is evident in the HMBC in the overlap of the proton region from 5.0-5.5 ppm and the carbon-13 from 25-50 ppm. The signals in this region that link the olefin carbons to the protons on the α-carbons relative to the olefins only match that of the same species (NB to NB and COE to COE), which further confirms that NMR is unable to detect NB to COE linkages within the films.

SI.9 – Detection Limits in NMR Analyses

NB-COE connections are not observed in Figure 3b for the pNB + pCOE HSQC spectrum, which is consistent with the concentration of these connections and the reported limit of detection for NMR analyses. While the coupling of ^{1}H signals to ^{13}C signals in ^{1}H-^{13}C HSQC experiments improves the limit of detection of ^{13}C experiments from ~1-10 mM to ~10-100 μM for a standard 5 mm NMR tube,[5–7] if COE repeat units were to add to every pNB chain in the pNB + pCOE, the concentration of NB-COE connections in a given NMR tube would be less than the ~10-100 μM limit of detection for ^{1}H-^{13}C HSQC. A pNB film has a mass of 2.5 mg, volume of 2 x 10^{-3} mL, molecular weight of 530 kDa (from Table 1), and is dissolved in 2 mL of $CDCl_3$ for NMR analysis, so the maximum concentration of NB-COE connections for a fully blocked sample is only ~2.4 μM inside the NMR tube:

$$\left(\frac{1\ mol\ NB - COE\ connections}{1\ mol\ chains}\right)\left(\frac{1\ mol\ chains}{530{,}000\ g\ chains}\right)\left(\frac{2.5\ \times\ 10^{-3}\ g\ chains}{0.002\ L\ solution}\right) = 2.4\ \mu M \quad (S1)$$

2.4 μM is below the estimated limit of detection for ^{1}H-^{13}C HSQC. Further mixing of repeat units within the film would need to be significant enough to have ~4-40x the NB-COE connections per chain to be observable in NMR.

SI.10 – Top Layer Formation in Layered Films of Varied Wettability

ATR-IR was used in Figure S8 to verify the presence of pNBF*n* layers on a pCOE bottom layer for the pCOE + pNBF*n* films presented in Table 2.

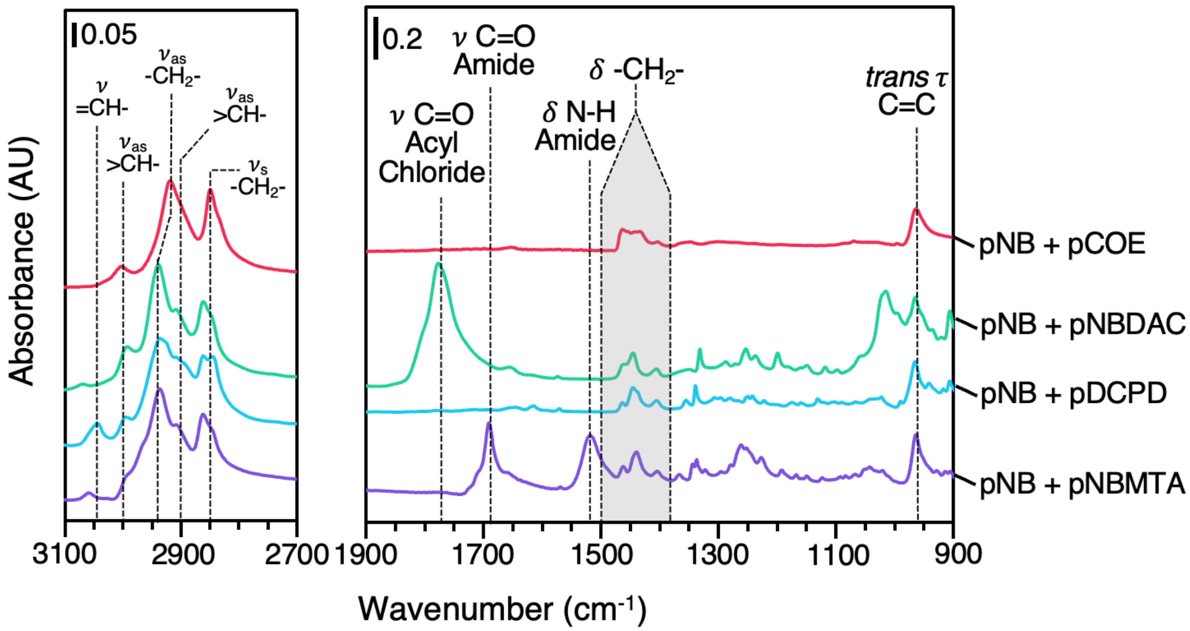

Figure S8. ATR-IR spectra of pNBF4, pNBF6, and pNBF8 top layers on a pCOE lower layer along with a pCOE single-layer spectrum. Spectra are normalized so all $\nu_{s/as}$ >CH-, =CH-, and -CH_2- areas are equivalent.

pCOE formation is demonstrated by the appearance of hydrocarbon stretching from 3100-2700 cm^{-1}, -CH_2- scissoring at ~1450 cm^{-1}, and *trans* olefin out-of-plane bending at 960 cm^{-1}. For the layered films, an additional collection of vibrations from 1300-1100 cm^{-1} often referred to as the perpendicular -CF_2- stretching region[8] verifies the presence of pNBF*n* layers on top of pCOE.

The other top layers displayed in Table 2 are synthesized on a pDCPD bottom layer instead of a pCOE bottom layer. ATR-IR spectra for pDCPD + pCOE and pDCPD + pNBDAC modified with hydroxylamine, arginine, and lysine solutions are shown in Figure S9.

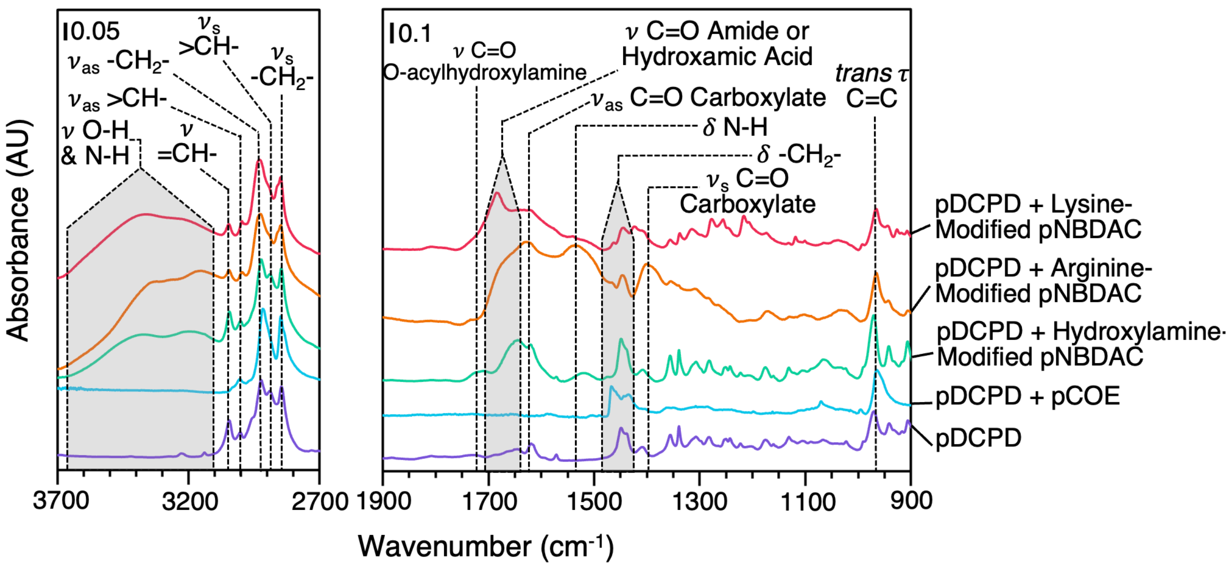

Figure S9. ATR-IR spectra of a pDCPD homopolymer, a pDCPD + pCOE two-layer polymer, and hydroxylamine-, arginine-, and lysine-modified pDCPD + pNBDAC two-layer polymer films. Spectra are normalized so all $\nu_{s/as}$ >CH-, =CH-, and -CH$_2$- areas are equivalent.

pDCPD shows similar hydrocarbon vibration peaks at 3100-2700 cm^{-1}, 1500-1400 cm^{-1}, and 960 cm^{-1} to pNB and pCOE spectra. pDCPD also shows additional =CH- stretching beyond 3000 cm^{-1} and a C=C stretch at 1616 cm^{-1} from non-metathesized olefins in the cyclopentyl ring substituents. The pDCPD + pCOE spectrum is comparable to the pCOE spectrum in Figure S8, confirming the presence of a thick pCOE layer on pDCPD. No pDCPD is observed in the spectrum due to the several micron thick information depth of ATR-IR.[9] Successful polymerization and modification of the pNBDAC top layer to the hydroxamic acid product is evident by the ν C=O of the hydroxamic acid (also known as the N-acylhydroxylamine moiety) at 1646 cm^{-1} and a smaller peak for the O-acylhydroxylamine minor product at 1711 cm^{-1}. Arginine- and lysine-modified pNBDAC top layers

show ν C=O and δ N-H vibrations signifying successful conversion to the amide products, and ν_{as} and ν_s C=O vibrations for the carboxylate groups present on the lysine and arginine modifiers that are incorporated into the films. A broad collection of peaks from 3600-3100 cm^{-1} are observed for O-H and N-H stretching of the modified acyl chloride products in all three of the modified pNBDAC top layers. The ν O-H and N-H signals for the arginine-modified film show local maxima at 3345 and 3178 cm^{-1}, which are both distinguished relative to the lysine- and hydroxylamine-modified films and are consistent with previous IR spectra for polymers containing guanidine groups.[10]

SI.11 – Surface Energy Estimations for Layered Films of Varied Wettability

Zisman plots were used to determine the critical surface tension values (γ_c) for the pCOE + pNBFn films used in this study.[11] The Zisman method is appropriate for these surface compositions as pCOE + pNBFn films do not contain strong polar or hydrogen bonding components,[12] and previous analyses with pNBFn homopolymers show that changes in contact angles by these fluorocarbon surfaces are well fit by the model proposed by Zisman.[13] Zisman plots for pCOE + pNBFn films using a series of n-alkane probe liquids are shown in Figure S10 along with the lines of best fit for each series.

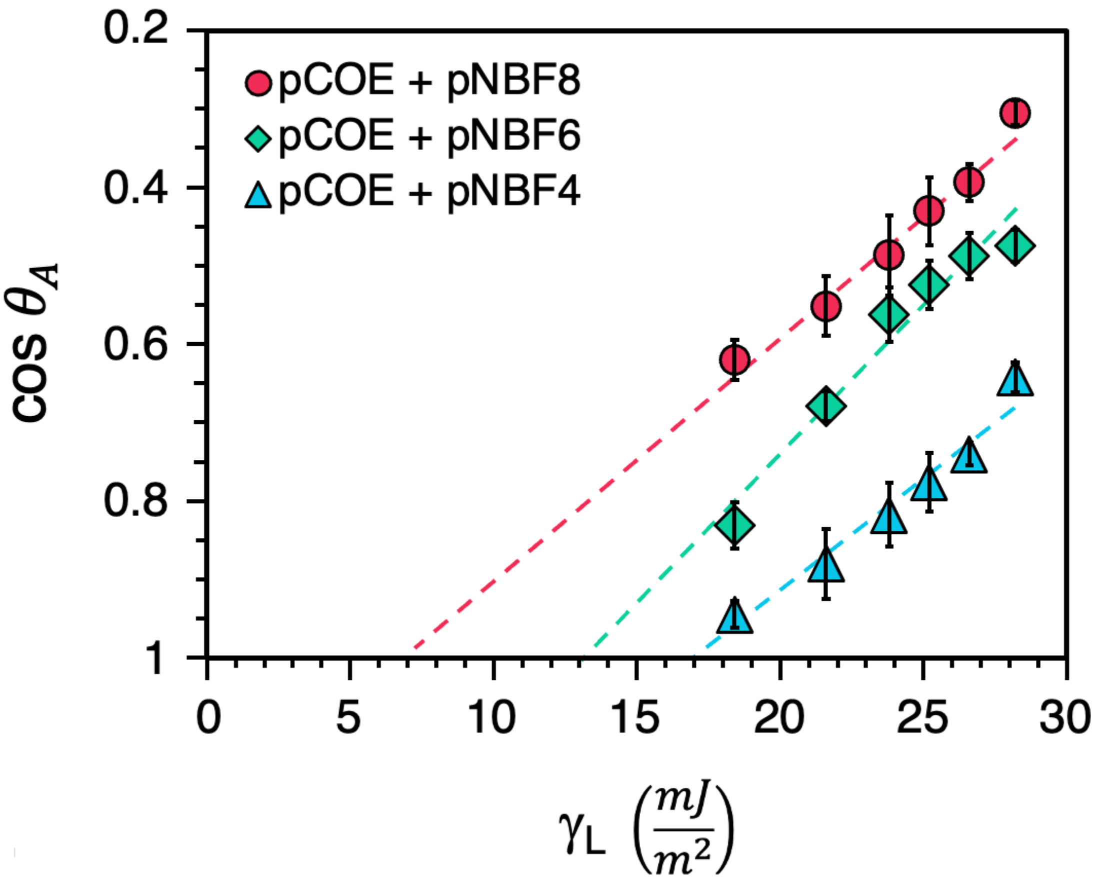

Figure S10. Zisman plot for pCOE + pNBF*n* (*n* = 4, 6, and 8) films. Data points were obtained from the advancing contact angles on pCOE + pNBF*n* films using even-numbered *n*-alkane probe liquids from *n*-hexane to *n*-hexadecane. Dashed lines represent the lines of best fit for each data series extrapolated to the point where $cos\theta_A = 1$.

The theory proposed by Zisman states that a linear extrapolation of the line of best fit using either *n*-alkanes or *n*-alcohols as probe liquids to the presumed point of complete wetting, $cos\theta = 1$, yields γ_c for the film.[11] For films containing only dispersive components of surface tension, γ_c should be approximately equivalent to the surface energy of the solid,[11] so $\gamma_s \approx 17$, 13, and 7 mJ m^{-2} for pCOE + pNBF4, pCOE + pNBF6, and pCOE + pNBF8 films, respectively.

n-Alkane probe liquids either mostly wet or completely wet the surfaces of the pCOE and modified pNBDAC top layers listed in Table 2, so the Owens-Wendt method[14] was instead used to obtain surface energy estimates for those films in Figure S11.

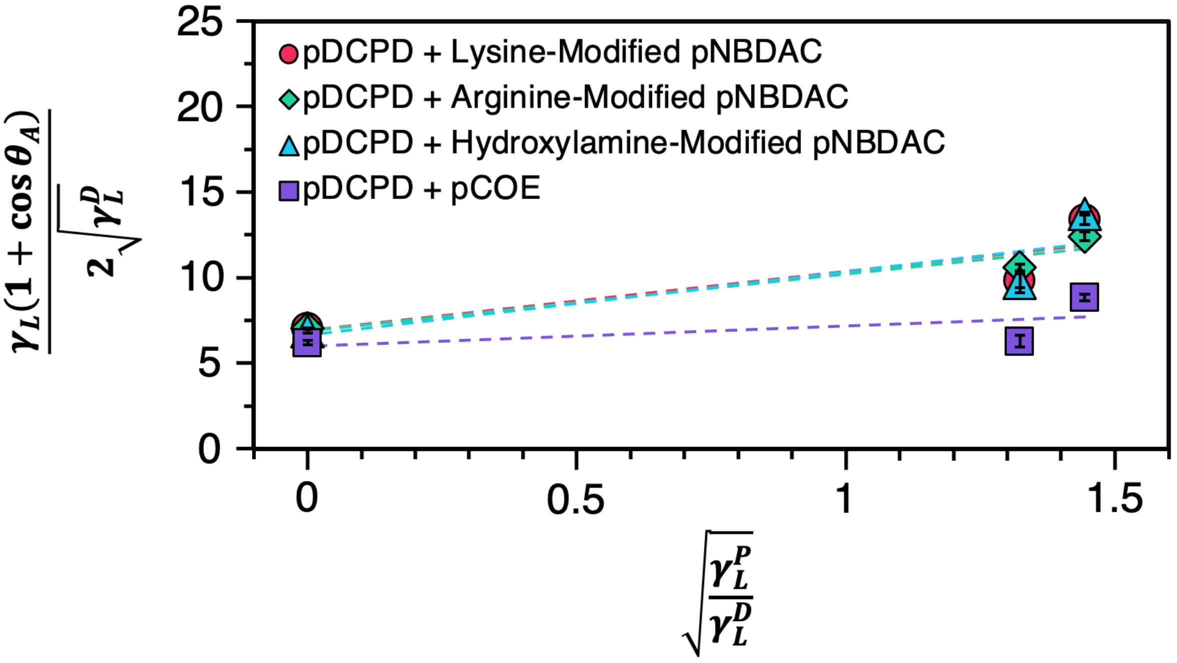

Figure S11. Owens-Wendt plot for films with pDCPD bottom layers and pCOE, lysine-modified pNBDAC, arginine-modified pNBDAC, or hydroxylamine-modified pNBDAC top layers. Diiodomethane, ethylene glycol, and glycerol were used as probe liquids.

An advantage of the Owens-Wendt method over the Zisman method is that the analysis yields values for both the dispersive (γ_s^D) and polar (γ_s^P) contributions of surface energy. By combining the contributions of Young,[15] Good and Girifalco,[16] and Fowkes[17] together, Owens and Wendt developed the following equation:

$$\frac{\gamma_L(1+\cos\theta)}{2\sqrt{\gamma_L^D}} = \sqrt{\gamma_s^D} + \sqrt{\gamma_s^P}\sqrt{\frac{\gamma_L^P}{\gamma_L^D}} \quad (S4)$$

Thus, when $\frac{\gamma_L(1+\cos\theta)}{2\sqrt{\gamma_L^D}}$ is plotted against $\sqrt{\frac{\gamma_L^P}{\gamma_L^D}}$, the slope and the y-intercept of the line of best fit would be $\approx \sqrt{\gamma_s^P}$ and $\sqrt{\gamma_s^D}$, respectively. γ_s^P values for the modified pNBDAC films were far greater than the pCOE film due to their more hydrophilic amino, guanidine, carboxylic acid, or hydroxamic acid substituents.

SI.12 – Impact of Spin Speed on pCOE Top Layer Thickness

The effect of COE monomer dispense spin speed on pCOE layer thickness when (1) dispensed as a single monomer solution on a silicon substrate and (2) after a NB dispense to form a layered polymer film on silicon is shown in Figure S12.

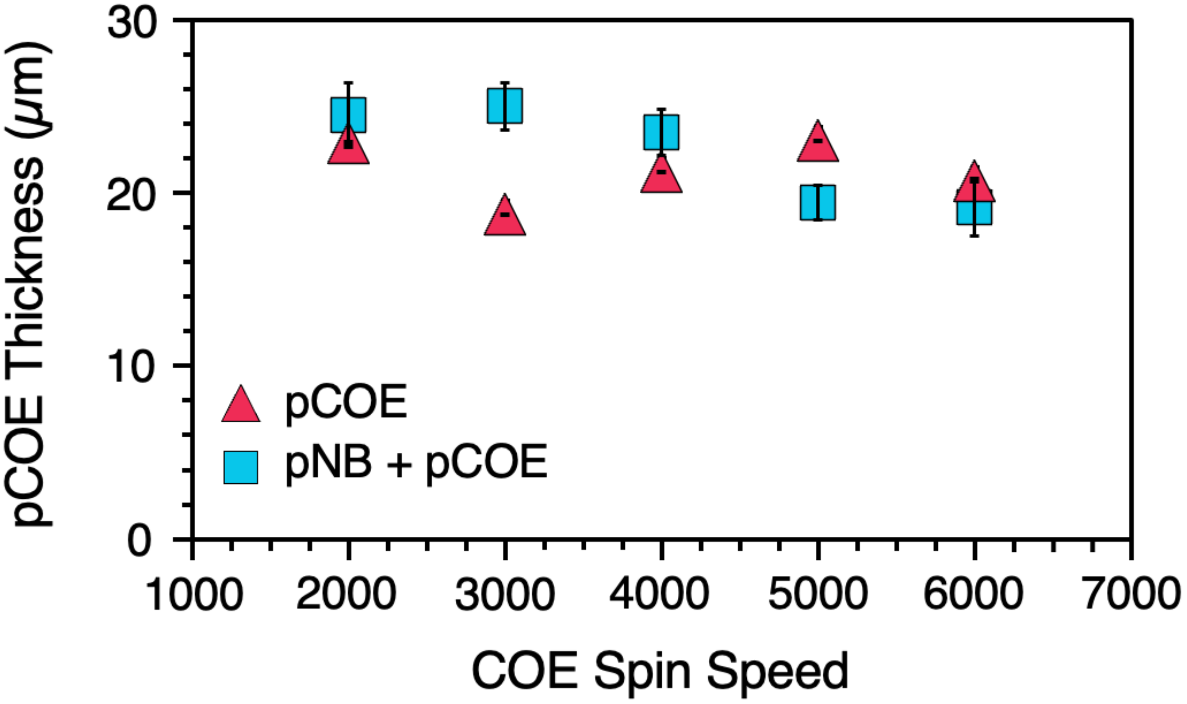

Figure S12. pCOE thickness as a homopolymer (pCOE) and as a top layer on pNB (pNB + pCOE) as a function of COE monomer dispense spin speed on a silicon substrate. Spin speed for the catalyst was 2000 RPM, and the spin speed for the NB monomer was 2000 RPM for the pNB + pCOE systems. The bottom layer thickness was 8 ± 2 µm for pNB in the pNB + pCOE systems.

While increasing spin speed using NBDAC as a monomer has previously resulted in a substantial decrease in thickness,[18] no such significant decrease was observed when pCOE was grown via scROMP. A similar trend is observed with the pNB + pCOE two-layer film thickness, where the pCOE top layer thickness remains ~20 µm as spin speed in increased from 2000 to 6000 RPM.

SI.13 –Top Layer Formation for Various Compositions on pNB

Top layer thicknesses of pCOE, pNBDAC, pDCPD, and pNBMTA on pNB are presented in Table 3, and ATR-IR spectra verifying the formation of the layered polymer films are shown in Figure S13.

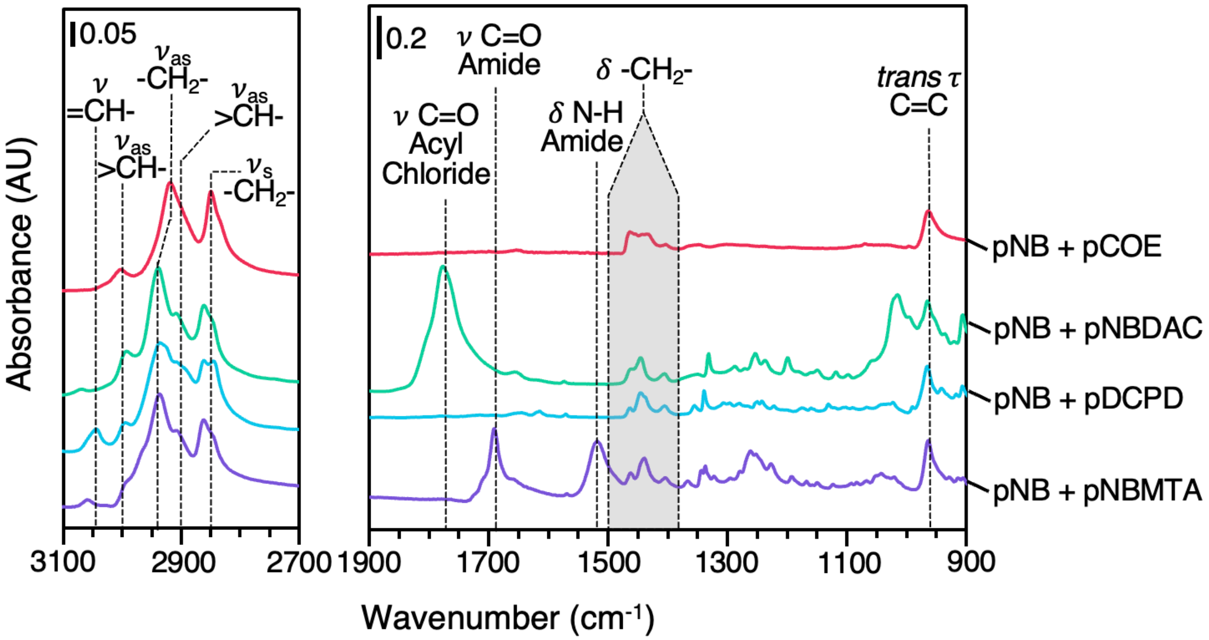

Figure S13. ATR-IR spectra of pNBMTA, pDCPD, pNBDAC, and pCOE top layers on a pNB lower layer. Spin speeds for all dispenses were 2000 RPM, and spectra are normalized so all $\nu_{s/as}$ >CH-, =CH-, and $-CH_2-$ areas are equivalent.

Successful polymerization of pNBMTA is verified through the presence of the ν C=O and δ N-H vibrations at 1690 and 1518 cm^{-1}, respectively, from the amide group attached to each cyclopentyl ring. pDCPD formation is confirmed by $\nu_{s/as}$ =CH- vibrations at 3044 and 2994 cm^{-1} from non-metathesized olefin groups in the cyclopentyl ring substituents that are not present in the pNB ATR-IR spectrum in Figure 6. ν C=O vibrations at 1776 cm^{-1} are associated with the presence of the two acyl chloride groups per NBDAC repeat and confirm successful polymerization of the pNBDAC top layer. pCOE is a completely hydrocarbon polymer and does not possess vibrations that appear at distinct wavenumbers from those in pNB, but stronger absorption peaks at 2918 and

2849 cm^{-1} that characterize pCOE are observed due to the larger ratio of -CH_2- to >CH- moieties in pCOE than pNB. The four spectra show greater contributions of the top layer than the pNB lower layer because the top layers are in general thicker than the approximately several micron information depth of ATR-IR.[9]

SI.14 – Magnified Lysine-Modified pNB + pDCPD + pNBDAC Spectrum

The ATR-IR spectrum of lysine-modified pNB + pDCPD + pNBDAC is shown in Figure 6; however, the C=O stretching region peaks are not sufficiently well-defined at the displayed scale. To better resolve the individual stretching peaks of interest, a magnified version of the lysine-modified pNB + pDCPD + pNBDAC spectrum is shown in Figure S14.

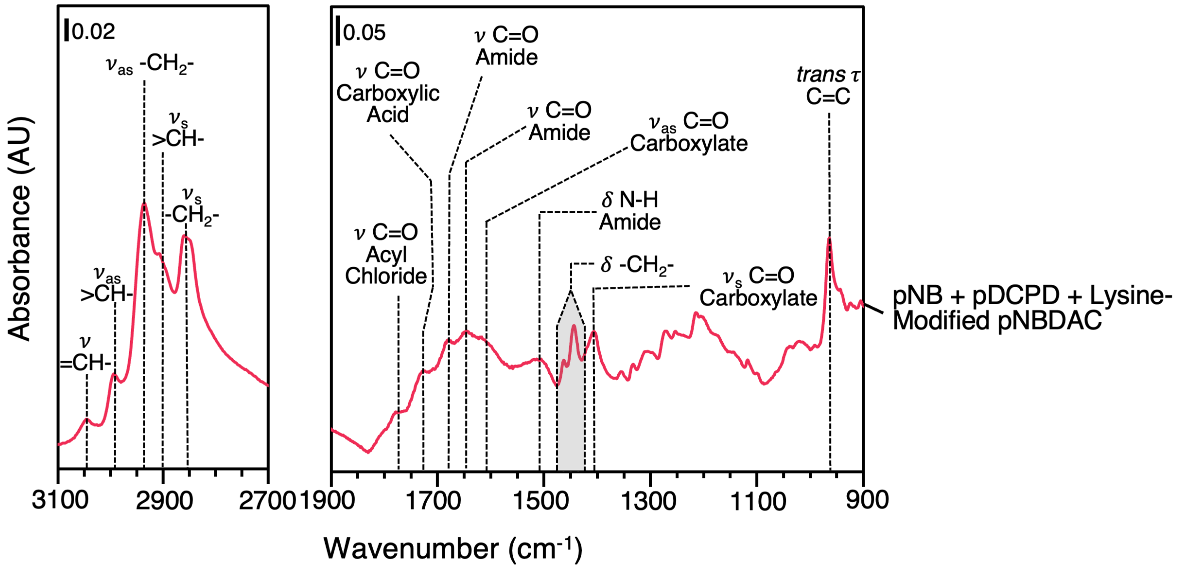

Figure S14. ATR-IR spectrum of lysine-modified pNB + pDCPD + pNBDAC at a smaller scale than shown in Figure 6.

Two minor υ C=O vibrations are observed at 1776 cm^{-1} and 1730 cm^{-1} for the unmodified acyl chloride and the carboxylic acid product formed through reaction with the solvent, respectively. Two major υ C=O vibrations associated with the formation of the amide products are observed at 1648 and 1680 cm^{-1} for the reaction of the acyl chloride groups with the two different amines on

a lysine molecule. While the water solvent is present at much higher concentrations (55 M) than the lysine (0.1 M), aminolysis occurs at a much faster rate than hydrolysis for acyl chlorides,[19] and therefore amide functional groups exist at greater concentrations throughout the film than carboxylic acid groups. C=O stretching vibrations for the carboxylic acid and carboxylate moieties present in the lysine and arginine modifiers are observed at 1730, 1608, and 1410 cm^{-1}.

SI.15 – Roughness Values for Certain Films in the Three-Layered Systems

Film roughness can impact values reported in contact angle measurements.[20,21] To better analyze potential roughness-based discrepancies in contact angles, roughness values obtained from profilometry are shown in Table S2.

Table S2. Film roughness values obtained from profilometry for certain polymers used in the three-layered system analysis. The lysine-modified pNBDAC film had to be taped to the silicon substrate using double sided tape as it tended to delaminate and curl after removal from the modifying solution as shown in Figure S6.

Film	Roughness (nm)
pNB (Pentane as Solvent)	2200
pNB (50/50 v/v % Pentane/DCM as Solvent)	340
Lysine-Modified pNBDAC	340
pDCPD + Lysine-Modified pNBDAC	150
pNB + pDCPD + Lysine-modified pNBDAC	3600

The use of pentane as a solvent increases roughness substantially for pNB relative to when pNB is synthesized using the 50/50 v/v % pentane/DCM mixture, presumably due to the inability of pentane to act as a good solvent for norbornene. Roughness values are also substantially different when lysine-modified pNBDAC is synthesized as a homopolymer, as the top layer in a two-layer polymer film, and as the top layer in a three-layer polymer film. The two-layer polymer film is the smoothest of the three films, which is consistent with its slightly elevated contact angle in Table 4.

SI.16 – pH Measurements for 0.1 $M_{(aq)}$ Solutions of Lysine and Arginine

pH measurements were obtained for 0.1 $M_{(aq)}$ lysine and arginine modification solutions in Table S3.

Table S3. pH measurements of aqueous lysine and arginine solutions.

Solution	pH
0.1 M Lysine (aq)	9.8
0.1 M Arginine (aq)	11.9

Lysine and arginine are zwitterions from $9 < pH < 10.5$ and $9 < pH < 12.5$,[22] respectively, so both are zwitterionic for the concentrations used in the pNBDAC modifications. Once bound through the neutral, more nucleophilic amine group, the lysine- and arginine-modified films are zwitterionic from $2 < pH < 10.5$ and $2 < pH < 12.5$, respectively.[22]

SI.17 – Contact Angles, Fluorescence Intensity, and Non-Fluorescent Confocal Images for Protein-Resistant Films

Advancing contact angles with water were obtained in Table S4 to analyze the effect of hydrophilicity on relative fluorescence intensity for the films shown in Figure 7 upon exposure to albumin.

Table S4. Advancing contact angles using water as a probe liquid and relative fluorescence intensity for the films in Figure 7. Relative fluorescence intensity was determined by measuring the green color pixel counts of the entire confocal image using GIMP software.

Film	θ_A H_2O (°)	Relative Fluorescence Intensity
pNB	152 ± 2	1
pDCPD	94 ± 1	0.29
pNB + pDCPD	96 ± 3	0.59
pNB + pDCPD + Octanol-Modified pNBDAC	82 ± 3	0.21
pNB + pDCPD + Lysine-Modified pNBDAC	< 15	0.02
pNB + pDCPD + Arginine-Modified pNBDAC	33 ± 3	0.04

Lower contact angles were in general associated with lower fluorescence intensities. Films with contact angles from 82-94° showed ~2-5x less protein adsorption than that of the strongly hydrophobic pNB, and films with contact angles of ≤ 33° showed ≥ 25x less protein adsorption than pNB exhibited. The large difference in fluorescence intensity between pDCPD and pNB + pDCPD cannot be attributed to differences in contact angles, however, as their contact angles do not statistically deviate from each other. Non-fluorescent analogues of the images in Figure 7 are shown in Figure S15 to determine if visual roughness impacted protein adsorption.

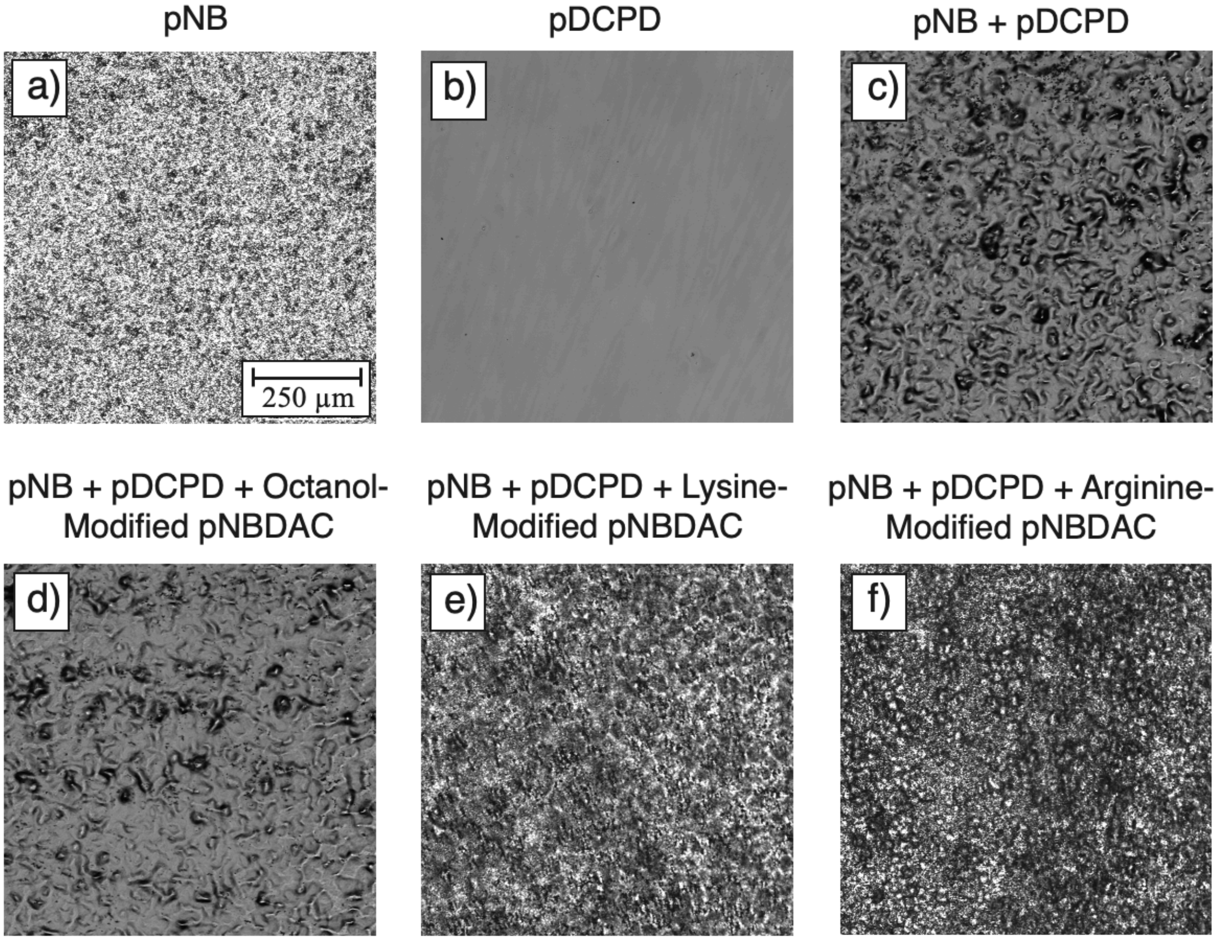

Figure S15. Confocal microscopy images of a) pNB and b) pDCPD single-layer films; c) a pNB + pDCPD two-layer film; and pNB + pDCPD + pNBDAC three-layer films modified with d) octanol, e) lysine, and f) arginine without fluorescence excitation.

pDCPD is by far the smoothest film of the six films tested in protein adsorption experiments, which may explain its seemingly low protein adsorption relative to pNB + pDCPD. The rougher pNB + pDCPD has more exposed surface area for protein binding per area of glass substrate, potentially yielding fluorescence values that are misleadingly high.

SI.18 – Formation of the pNB + pDCPD + (Octanol-Modified pNBDAC) Film

Successful modification of the pNBDAC layer to the octyl ester product is shown using ATR-IR in Figure S16 by the presence of the ν C=O at 1727 cm^{-1}, ν C-C-O at 1166 cm^{-1}, and ν O-C-C at 1041 cm^{-1}, and the lack of a stretching peak at 1774 cm^{-1} for ν C=O acyl chloride.

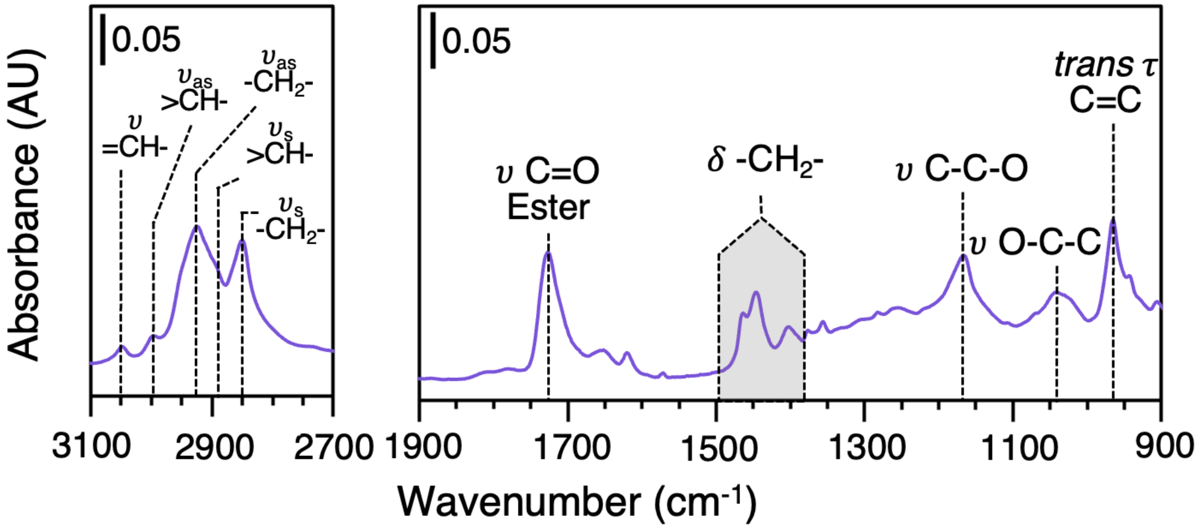

Figure S16. ATR-IR of an octanol-modified pNB + pDCPD + pNBDAC film.

ATR-IR compositional verification for other modified pNBDAC layers shown in Figure 7 are displayed in Figure 6 and S9.

References

(1) Silverstein, R. M.; Webster, F. X.; Kiemle, D. J. *Spectrometric Identification of Organic Compounds*, 7th ed.; John Wiley & Sons, Inc.: Hoboken, New Jersey, 2005.

(2) Suriboot, J.; Hu, Y.; Malinski, T. J.; Bazzi, H. S.; Bergbreiter, D. E. Controlled Ring-Opening Metathesis Polymerization with Polyisobutylene-Bound Pyridine-Ligated Ru(II) Catalysts. *ACS Omega* **2016**, *1* (4), 714–721. https://doi.org/10.1021/acsomega.6b00218.

(3) Stokes, G. G. On the Effect of the Internal Friction of Fluids on the Motion of Pendulums. *Trans. Camb. Philos. Soc.* **1851**, *9*.

(4) Einstein, A. On the Motion of Small Particles Suspended in Liquids at Rest Required by the Molecular-Kinetic Theory of Heat. *Ann. Phys.* **1905**, *17*, 549–560.

(5) Lee, J. H.; Okuno, Y.; Cavagnero, S. Sensitivity Enhancement in Solution NMR: Emerging Ideas and New Frontiers. *J. Magn. Reson.* **2014**, *241*, 18–31. https://doi.org/10.1016/j.jmr.2014.01.005.

(6) Schulze-Sünninghausen, D.; Becker, J.; Luy, B. Rapid Heteronuclear Single Quantum Correlation NMR Spectra at Natural Abundance. *J. Am. Chem. Soc.* **2014**, *136* (4), 1242–1245. https://doi.org/10.1021/ja411588d.

(7) Bingol, K.; Brüschweiler, R. Multidimensional Approaches to NMR-Based Metabolomics. *Anal. Chem.* **2014**, *86* (1), 47–57. https://doi.org/10.1021/ac403520j.

(8) Fukushima, H.; Seki, S.; Nishikawa, T.; Takiguchi, H.; Tamada, K.; Abe, K.; Colorado, R.; Graupe, M.; Shmakova, O. E.; Lee, T. R. Microstructure, Wettability, and Thermal Stability of Semifluorinated Self-Assembled Monolayers (SAMs) on Gold. *J. Phys. Chem. B* **2000**, *104* (31), 7417–7423. https://doi.org/10.1021/jp0003499.

(9) Milosevic, M. Internal Reflection and ATR Spectroscopy. *Appl. Spectrosc. Rev.* **2004**, *39* (3), 365–384.

(10) Răducanu, C. E.; Dobre, T.; Mihăiescu, D. E.; Moroşan, A.; Jidveian, R.; Cioroiu Tîrpan, D. R.; Vasiliu, A. D.; Gogoaşă, C. I.; Pârvulescu, O. C.; Trică, B. Synthesis of Guanidine and Its Deposition on Bacterial Cellulose as Green Heterogeneous Catalyst for Transesterification to Methyl Esters. *Energies* **2024**, *17* (6), 1344. https://doi.org/10.3390/en17061344.

(11) Fox, H. W.; Zisman, W. A. The Spreading of Liquids on Low Energy Surfaces. I. Polytetrafluoroethylene. *J. Colloid Sci.* **1950**, *5* (6), 514–531. https://doi.org/10.1016/0095-8522(50)90044-4.

(12) Zisman, W. A. Relation of the Equilibrium Contact Angle to Liquid and Solid Constitution. In *Contact Angle, Wettability, and Adhesion*; Advances in Chemistry; American Chemical Society, 1964; Vol. 43, pp 1–51. https://doi.org/10.1021/ba-1964-0043.ch001.

(13) Vasuta, M. P.; Parkerson, Z. J.; Oddo, T. D.; Rogers, B. R.; Jennings, G. K. Fluorocarbon Minimization Via Semifluorinated Copolymer Films by Combining Spin Coating and Ring-Opening Metathesis Polymerization. *Langmuir* **2025**, *41* (10), 6931–6943. https://doi.org/10.1021/acs.langmuir.4c05253.

(14) Owens, D. K.; Wendt, R. C. Estimation of the Surface Free Energy of Polymers. *J. Appl. Polym. Sci.* **1969**, *13* (8), 1741–1747. https://doi.org/10.1002/app.1969.070130815.

(15) Young, T. An Essay on the Cohesion of Fluids. *Abstr. Pap. Print. Philos. Trans. R. Soc. Lond.* **1805**, *1*, 171–172. https://doi.org/10.1098/rspl.1800.0095.

(16) Girifalco, L. A.; Good, R. J. A Theory for the Estimation of Surface and Interfacial Energies. I. Derivation and Application to Interfacial Tension. *J. Phys. Chem.* **1957**, *61* (7), 904–909. https://doi.org/10.1021/j150553a013.
(17) Fowkes, F. M. Attractive Forces at Interfaces. *Ind. Eng. Chem.* **1964**, *56* (12), 40–52. https://doi.org/10.1021/ie50660a008.
(18) Parkerson, Z. J.; Prozorovska, L.; Vasuta, M. P.; Oddo, T. D.; Jennings, G. K. Simultaneous Spin Coating and Ring-Opening Metathesis Polymerization for the Rapid Synthesis of Polymer Films. *ACS Appl. Mater. Interfaces* **2024**, *16* (13), 16754–16766. https://doi.org/10.1021/acsami.4c00211.
(19) Bentley, T. W.; Llewellyn, G.; McAlister, J. A. S(N)2 Mechanism for Alcoholysis, Aminolysis, and Hydrolysis of Acetyl Chloride. *J. Org. Chem.* **1996**, *61* (22), 7927–7932. https://doi.org/10.1021/jo9609844.
(20) Wenzel, R. N. Resistance of Solid Surfaces to Wetting by Water. *Ind. Eng. Chem.* **1936**, *28* (8), 988–994. https://doi.org/10.1021/ie50320a024.
(21) Cassie, A. B. D. Contact Angles. *Discuss. Faraday Soc.* **1948**, *3* (0), 11–16. https://doi.org/10.1039/DF9480300011.
(22) Fiori, F.; Cossu, F. L.; Salis, F.; Carboni, D.; Stagi, L.; De Forni, D.; Poddesu, B.; Malfatti, L.; Khalel, A.; Salis, A.; Casula, M. F.; Anedda, R.; Lori, F.; Innocenzi, P. In Vitro Antiviral Activity of Hyperbranched Poly-L-Lysine Modified by L-Arginine against Different SARS-CoV-2 Variants. *Nanomaterials* **2023**, *13* (24), 3090. https://doi.org/10.3390/nano13243090.
